# Mind–body training outperforms other physical activities in reducing frailty and enhancing quality of life in older adults: a network meta-analysis

**DOI:** 10.3389/fpubh.2025.1578791

**Published:** 2025-07-14

**Authors:** Guangwen Liu, Renkai Ge, Huiling Zhu

**Affiliations:** School of Physical Education and Health, East China Jiaotong University, Nanchang, China

**Keywords:** physical activity, frailty, activities of daily living, quality of life, older adults

## Abstract

**Background:**

Physical activity is an effective strategy for treating and intervening in frailty syndrome, which leads to functional decline, increased hospitalization rates, and heightened mortality risks among older adult people. However, the relative effectiveness of different types of physical activity modalities in mitigating frailty and enhancing activities of daily living (ADLs) and quality of life (QoL) remains insufficiently explored. This network meta-analysis quantified and compared the effects of different physical activities on frailty, ADLs, and QoL, providing an evidence base for targeted interventions.

**Methods:**

Following the PRISMA-NMA guidelines, we conducted systematic searches of PubMed, Embase, and CNKI to identify 35 randomized controlled trials (N = 2,905) between January 2000 and August 2024. We employed the frequency science network meta-analysis model to quantify the relative efficacy of various physical activities in enhancing frailty management, ADLs, and QoL in older people.

**Results:**

The analysis revealed that Mind–body training (e.g., Taiji, Baduanjin), a traditional Chinese exercise that integrates gentle body movements with breath regulation, significantly alleviated debilitating conditions (SMD = −0.71, 95% CI: −1.22 to −0.21) and markedly improved quality of life (SMD = 1.02, 95% CI: 0.89 to 1.15). This modality proved to be the most effective of all the interventions studied. Aerobic training was particularly beneficial for enhancing the ability to perform ADLs (SMD = 0.89, 95% CI: 0.06 to 1.72). Although mixed physical activity exhibited overall efficacy across all health indicators (*p* < 0.05), its benefits did not exceed those of mind–body training. Further analysis indicated that the intervention regimen consisting of 50–60 min of training per session, 2–3 times per week, yielded the most significant improvements.

**Conclusion:**

This study employed network meta-analysis to compare the effects of various physical activities on frailty, ADLs, and QoL in older adults. The findings suggest that both mental and physical training significantly ameliorate frailty and enhance QoL, whereas aerobic training enhances daily living capabilities. Clinical interventions should prioritize tailored mental and physical training, considering individual differences. Future studies should focus on long-term effects and dose–response relationships to optimize frailty management and health promotion in older adult individuals.

## Introduction

Frailty is a multifactorial clinical syndrome characterized by a decline in multiple physiological systems in older adults, leading to increased susceptibility to external stressors (1). This condition reflects diminished physical reserves and a decline in psychological and social functioning, which heightens vulnerability to stressors (2). Frailty is characterized by reduced physical activity, decreased muscle strength, slower gait speed, increased fatigue, and involuntary weight loss (3). As the global population ages, the prevalence of frailty among community-dwelling older individuals is increasing. The latest data revealed that frailty affects between 10 and 25% of community-dwelling older people globally. The prevalence of frailty among people aged 60 years and older is increasing significantly worldwide, particularly in low- and middle-income countries (4). The prevalence of frailty among people over 60 years of age is increasing significantly worldwide, especially in low- and middle-income countries. This syndrome not only poses a significant health risk to affected individuals but also places considerable pressure on public health systems (5). As frailty worsens, it leads to higher rates of hospitalization, increased disability risk, and diminished quality of life, thus highlighting the urgent need for its prevention and management as a global public health priority (6).

Although frailty is associated primarily with reduced physical activity and muscle strength, its etiology involves complex neural and muscular mechanisms. Age-related deterioration in both the central and peripheral nervous systems is evident in the reduced number of motor neurons, slowing of nerve conduction, and decreased motor unit recruitment, which affects the efficiency of muscle activation and contraction. These results lead to diminished muscle strength and motor function (7). This phenomenon affects the efficiency of muscle activation and contraction, leading to diminished strength and impaired motor function (8, 9). The degeneration of motor neurons commences around the age of 60 and intensifies with advancing age (10), leading to a continuous decline in muscle mass and strength (11, 12). The loss of motor neurons is pronounced in the lower extremities, with healthy older adults having approximately 50% fewer motor neurons by the age of 70 than their younger counterparts do, further exacerbating the deterioration of muscle strength and increasing weakness (13, 14). Moreover, the atrophy of fast-contracting muscle fibers exacerbates symptoms such as decreased physical performance and unsteady gait (15). Together, these mechanisms of neurological and muscular degeneration contribute significantly to the debilitating symptoms observed in older adults, including decreased physical performance, increased fatigue, and unsteady gait. However, targeted interventions addressing these neurological and muscular degenerative processes can not only mitigate debilitating symptoms but also slow their progression, thus significantly enhancing the quality of life of older adult individuals (16). In light of this, evaluating physical activity as a therapeutic strategy for frailty has gained increasing research interest. Despite growing recognition of physical activity as a beneficial intervention for frailty, current research lacks comprehensive comparative analyses of various exercise modalities. Evidence remains fragmented, often focusing narrowly on single exercise types or constrained by methodological inconsistencies.

Consequently, systematic and rigorous evaluations to clarify the relative effectiveness of diverse physical activity interventions remain essential. Therefore, this study systematically compares different physical activity modalities—Mind–body training, aerobic training, strength training, and mixed physical activities—on frailty, activities of daily living (ADLs), and quality of life (QoL) among older adults, aiming to provide robust evidence to inform tailored and effective intervention strategies.

## Methods

### Research program

This study was conducted in accordance with the Cochrane Collaboration manual and the extended PRISMA-NMA (Preferred Reporting Items for Systematic Reviews and Meta-Analyses incorporating Network Meta-Analyses) guidelines (Appendix 1). As the analyses were based exclusively on previously published data, neither ethical approval nor patient consent was required.

### Search strategy

The data search spanned the CNKI, PubMed, Embase, Cochrane Library, and Web of Science databases from January 2000 to August 2024. It employs both medical subject headings (MeSH) and keywords (17), with a detailed strategy outlined in Appendix 2, focusing on terms including physical activity, older adult, frailty, and randomized controlled trials (RCTs).

### Inclusion and exclusion criteria

The inclusion criteria were as follows: (a) studies that compared various physical activities (aerobic training, strength training, Mind–body training, mixed physical activity) (Appendix 4) or those comparing physical activity against a control group (no systematic training) in older adults aged 60 years and older. (b) Included studies were those reporting on outcomes of frailty, activities of daily living (ADLs), and quality of life (SF-36) (Appendix 3) in the format of randomized controlled trials.

The exclusion criteria were nonrandomized controlled trials, acute interventions, and studies that failed to report metrics of frailty improvement.

### Outcome definitions

Considering the inherent variability across studies, we pre-specified eligible instruments to ensure clarity and consistency in the measurement of primary outcomes. Frailty was systematically assessed using widely accepted, validated scales, including the Frailty Phenotype, Clinical Frailty Scale, FRAIL Scale, Study of Osteoporotic Fractures (SOF) criteria, Tilburg Frailty Indicator, Short Physical Performance Battery (SPPB), Frailty Index, and the Edmonton Frailty Scale (18). Activities of daily living (ADLs) were measured using the Barthel Index, Katz ADL scale, and Lawton Instrumental Activities of Daily Living (IADL) scale (19). Quality of life (QoL) outcomes were evaluated through standardized instruments such as the 36-Item Short Form Health Survey (SF-36), the Short Form-12 (SF-12), EuroQol-5 Dimension (EQ-5D), and the Short Form-8 (SF-8) (20). Comprehensive details regarding these assessment tools, including scoring systems and interpretation criteria, are provided in Appendix 3a.

Due to variations in measurement tools utilized across studies, we employed standardized mean differences (SMD) to integrate and synthesize the data, ensuring robustness and comparability of results across diverse assessment scales (21–23).

In this systematic review, we clearly defined our research question according to the PICOS framework as follows: the target population consisted of community-dwelling or institutionalized older adults aged ≥ 60 years; interventions included various physical activity modalities, specifically mind–body training (e.g., Tai Chi, Baduanjin, mindfulness-based practices), aerobic training, strength training, and mixed physical activities; the comparators involved standard care, no structured intervention, or alternative physical/social activities; the primary outcomes assessed were frailty (measured using standardized instruments), activities of daily living (ADLs), and quality of life (QoL); and we only included randomized controlled trials (RCTs) as our designated study designs (24).

### Data extraction

Data extraction was independently performed by two researchers (G.W.L. and H.L.Z.) who independently screened the literature and compiled data from the final studies included into a standardized spreadsheet in Excel, as detailed in Appendix 5. The extracted information included the following: (1) authors and year of publication; (2) data related to subject characteristics (e.g., sample size, age, sex, and pre- and post-debilitation changes); and (3) details of pre- and post-debilitation interventions. Discrepancies in categorizing the interventions in each study were resolved through discussion, which involved a third-party researcher when necessary. In this systematic review, two researchers independently assessed all studies based on the extracted information (G.W.L. and H.L.Z.). A third reviewer (R.K.G.) was consulted to resolve any disagreements concerning study inclusion.

### Risk of bias assessment

The risk of bias in randomized controlled trials was independently evaluated by two reviewers (G.W.L. and H.L.Z.) via the revised Cochrane Risk of Bias, Version 2 (RoB 2) tool (25). Discrepancies were addressed through discussion, and in cases where consensus was not achievable, a third reviewer (R.K.G.) made the final decision.

### Data analysis

The transmissibility assumption is pivotal and fundamental in network meta-analysis (26). We evaluated the distribution of potential effect modifiers by contrasting various intervention modalities to substantiate this hypothesis. The factors potentially influencing outcomes included participants’ age and sex, as well as the duration and frequency of the intervention (27–29).

In this study, we used a systematic evaluation and network meta-analysis to ascertain the impact of different physical activities on ameliorating frailty among older adults using STATA 15.1 and the “netmeta 1.5–0” package of R software (30). Using STATA, we constructed a direct comparison network, where each point represents a type of intervention. The node’s size indicates the number of corresponding studies, and the connecting lines depict the direct comparisons between interventions (31). Data extraction encompassed the differences in means at baseline and endpoints, along with their standard deviations. In cases where standard deviations were unreported, they were estimated using standard errors, 95% confidence intervals, and interquartile ranges, with necessary transformations applied (21). Meta-analysis was conducted using the “netmeta” package in a random effects network framework, assessing heterogeneity with τ^2^ and I^2^ statistics (32). The coherence between direct and indirect evidence was evaluated using both global and local methods (33, 34) (Appendix 9). Discrepancies were statistically examined and documented through z scores and *p* values (Appendix 8), considering *p* < 0.05 as statistically significant (35, 36). In addition, we visually summarized the hierarchy of the effects of all exercise intervention modalities on frailty through the frequency ranking method and illustrated these effects in forest plots compared with those of the control group. This comprehensive analytical approach deepens our understanding of the differential effects of various exercise modalities. This study provides a robust scientific foundation for devising effective interventions for debilitating conditions in older adults.

## Results

### Literature selection

A comprehensive search yielded 443 documents, with 183 excluded initially due to duplication. After reviewing the titles and abstracts, we further excluded 63; an additional 48 were not retrievable; finally, 149 studies underwent full text screening.

**Figure 1:**
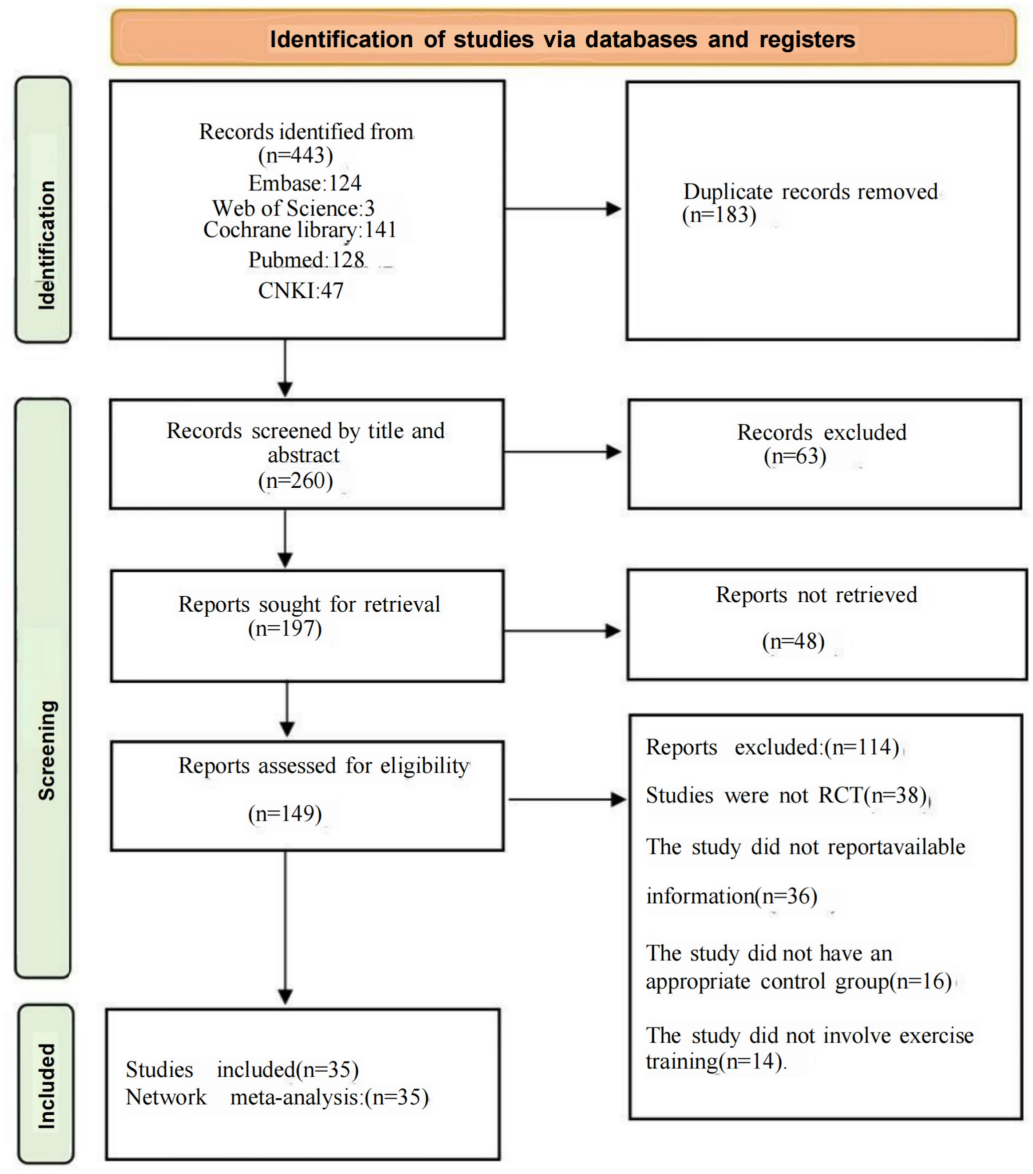
Literature review flowchart. Exercise frailty, RCT, randomized controlled trial.

After full-text screening, 114 studies were excluded for the following reasons: 38 studies were not randomized controlled trials, 36 studies failed to report appropriate outcomes or lacked analyzable data, 16 studies lacked a suitable control group, and 14 studies did not adhere to the intervention types specified in this study. Finally, 35 studies were included in our network meta-analysis, and the details of these exclusions and screenings are depicted in Figure 1.

### Characteristics of the included studies

A total of 2,905 participants aged 65 years and above were involved in our study, with a significantly higher number than that of male participants (female participants: 1772, 61% vs. male participants: 1133, 39%). Most of the studies were conducted in China (*N* = 16, 45.7%). The mean duration of the interventions for the frail participants was 18.1 weeks, with a frequency ranging from 2–7 sessions per week, and each session lasted between 20–80 min.

**Figure 2:**
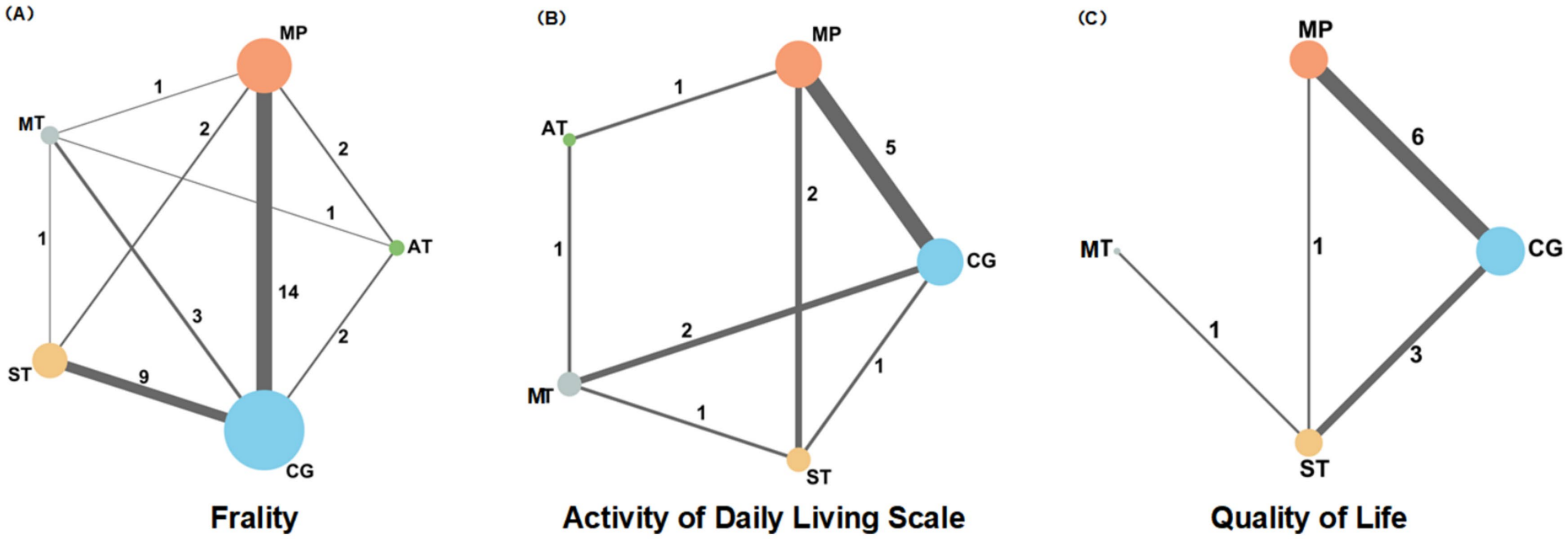
A network diagram showing the impact of different types of physical activity (MT, MP, AT, ST, CG) on frailty **(A)**, daily activities **(B)**, and quality of life **(C)**, in older adults.

Among the included studies, 14 were trials of mixed physical activities, while the remainder focused on single activity types. Thirty-five studies reported debilitating outcomes; aerobic and Mind–body training were discussed in 5 and 6 articles, respectively, while strength training and mixed activities were featured in 12 and 19 articles, respectively. An additional 13 studies reported outcomes related to ADLs, and 11 discussed qualities of life-related outcomes. The mesh diagram of these findings is presented in Figure 2. The demographic characteristics of the included studies are summarized in Appendix 5, and forest plots and funnel plots for all outcomes are presented in Appendices 7, 10.

### Risk of bias in the results

**Figure 3:**
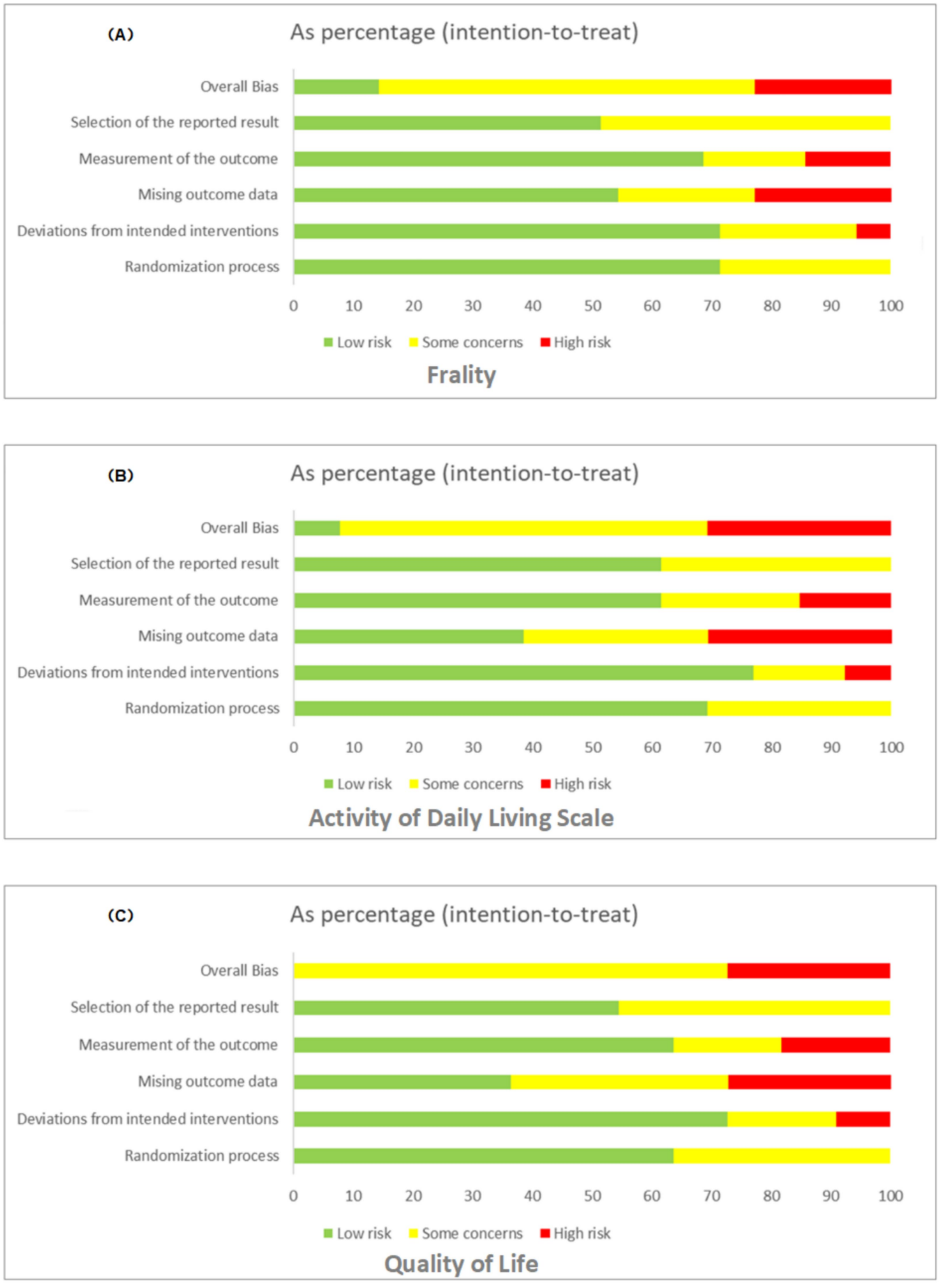
Bias assessment for frailty **(A)**, ADLs **(B)**, and QoL **(C)**, displaying risk levels (low, some concerns, high) across various factors.

The results from RoB 2 indicated that for the outcomes of frailty, ADLs, and quality of life assessments, the studies exhibited a high risk in 8.5, 12.2, and 15.7% respectively; some risk concerns in 25.4, 28.9, and 33.1%, respectively; and a low risk in 66.1, 58.9, and 51.2%, respectively. Overall, we judged the results of the frailty and activities of daily living outcomes to have some risk of bias, particularly regarding outcome measures and missing data. Where unadjusted registry outcome scores were reported, the risk of selective reporting was deemed low. As no instances of selective non-reporting were noted, no such bias risk was identified. The detailed process of risk assessment is shown in Figure 3 (Appendix 6, Figures S1–S3 for rationale).

### Network meta-analysis

This study evaluated the relative effectiveness of various physical activity interventions on three major health metrics, FP, ADLs, QoL in older adults, using a network meta-analysis (NMA). A comprehensive review of 35 randomized controlled trials (RCTs) (*N* = 2,905), revealed differential efficacy across these interventions in enhancing the specified health indicators. The detailed findings are outlined below:

### Frailty

**Figure 4:**
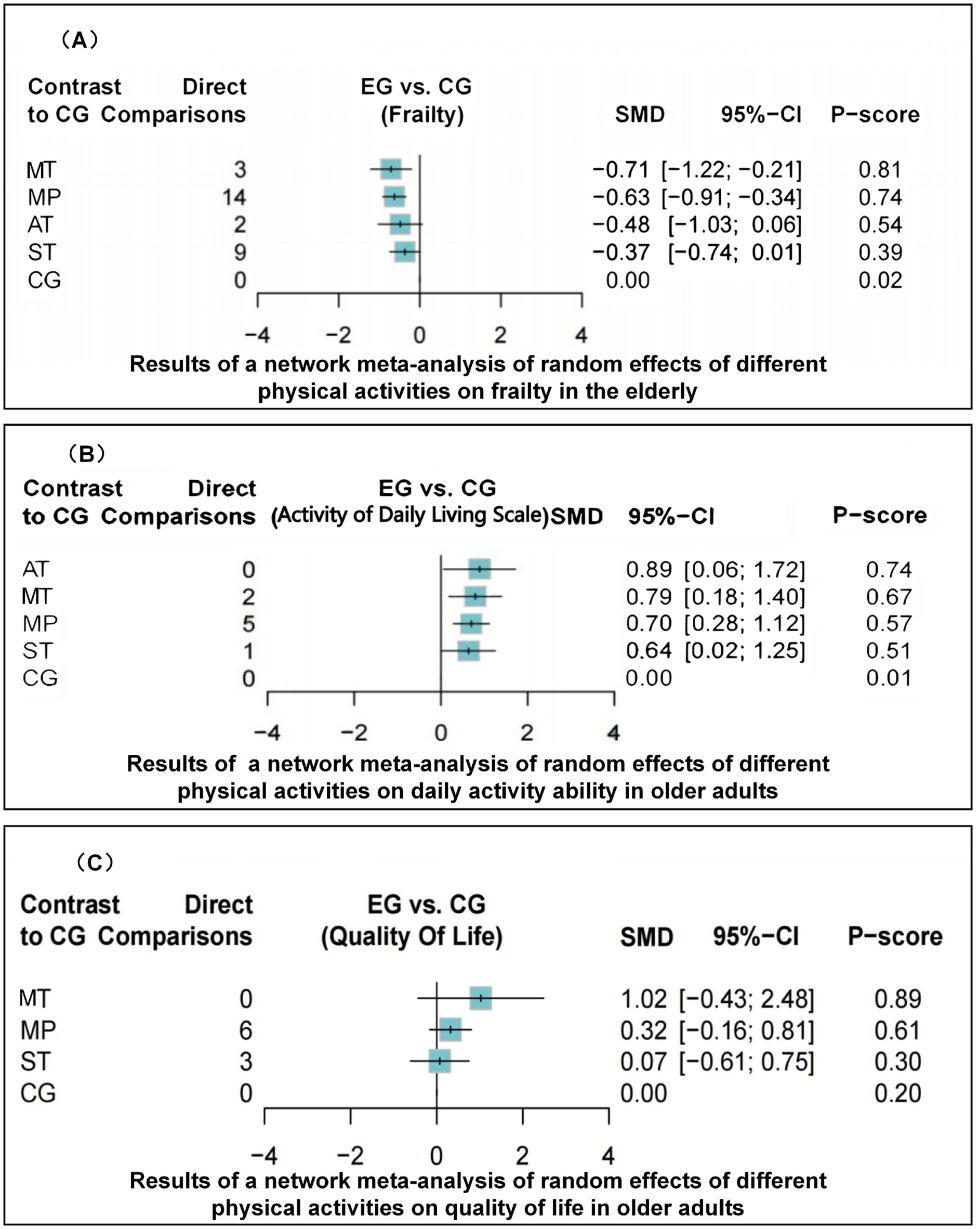
Forest plot of network meta-analysis outcomes showing effect sizes (SMD) for different physical activities (MT, MP, AT, ST, CG) on frailty **(A)**, ADLs **(B)**, and QoL **(C)** in older adults.

The dataset included results from 35 studies (N = 2,905), where the reduction in frailty was most pronounced with the combination of Mind–body training (MT). According to the data from Figures 2A, 4A, Mind–body training (MT) notably decreased the level of frailty in older adults relative to the control group (CG), achieving a standardized mean difference (SMD) of −0.71 (95% CI: −1.22 to −0.21), and a *p*-value of 0.81. Mixed physical activity (MP) followed, with an SMD of −0.63 (95% CI: −0.91 to −0.34) and a *p*-value of 0.81. Aerobic training (AT) and strength training (ST) displayed lesser effects, with SMDs of −0.48 (95% CI: −1.03 to 0.06) and −0.37 (95% CI: −0.74 to 0.01), and *p*-value of 0.54 and 0.39, respectively. The control group (CG), lacking systematic intervention, exhibited the least improvement (SMD = 0.00, *p*-value 0.02). As shown in Table 1, Mind–body training achieved the highest *p*-value, suggesting it surpassed other interventions in 81% of comparisons and was the most effective method to ameliorate frailty in the older adult.

**Table 1:** League table of standardized mean differences for frailty interventions.

MT	0.17 (−0.98; 1.32)	0.15 (−1.00; 1.30)	−0.11 (−1.31; 1.09)	**−1.02 (−1.72; −0.33)**
−0.08 (−0.62; 0.45)	MP	−0.11 (−0.93; 0.70)	−0.06 (−0.94; 0.83)	**−0.64 (−0.96; −0.32)**
−0.23 (−0.88; 0.43)	−0.15 (−0.70; 0.40)	AT	^.^	−0.25 (−1.08; 0.58)
−0.34 (−0.93; 0.24)	−0.26 (−0.70; 0.17)	−0.12 (−0.76; 0.53)	ST	−0.29 (−0.72; 0.14)
**−0.71 (−1.22; −0.21)**	**−0.63 (−0.91; −0.34)**	−0.48 (−1.03; 0.06)	−0.37 (−0.74; 0.01)	CG

The upper triangle reports estimates from direct head-to-head trials. A ‘.’ denotes the absence of such direct evidence. The lower triangle presents frequentist network meta-analysis results, integrating both direct and indirect comparisons across the treatment network. Bolded values indicate statistical significance, as their confidence intervals do not contain zero. The light green area in the upper triangle reports estimates from direct head-to-head trials, with “.” denoting the absence of such direct evidence. The yellow area in the middle ranks the standardized mean differences for various interventions, while the orange area in the lower triangle presents the network meta-analysis results, integrating both direct and indirect comparisons.

### Activities of daily living

Across 13 studies (*N* = 1,045), improvements in ADLs were optimally attained through aerobic training (AT) (Figure 2B). The SMD was 0.89 (95% CI: 0.06 to 1.72), with a *p*-value of 0.74, suggesting superiority over other interventions in 74% of cases. Mind–body training (MT) and mixed physical activity (MP) also significantly increased the ADLs, with SMDs of 0.79 (95% CI: 0.18 to 1.40) and 0.70 (95% CI: 0.28 to 1.12), and *p*-value of 0.67 and 0.57, respectively (Figure 4B). The impact of strength training (ST) was relatively modest (SMD = 0.64, 95% CI: 0.02 to 1.25) but still exceeded that of the control group (*p*-value = 0.51). According to Table 2, aerobic training emerged as the most effective for improving ADLs, with a *p*-value of 0.74.

**Table 2:** League table of standardized mean differences for ADL interventions.

MT	^.^	0.04 (−0.98; 1.06)	−0.07 (−1.11; 0.98)	0.83 (0.06; 1.59)
0.09 (−0.57; 0.75)	MP	−0.34 (−1.37; 0.69)	−0.02 (−0.81; 0.76)	0.76 (0.30; 1.22)
−0.10 (−0.89; 0.69)	−0.19 (−0.99; 0.60)	AT	^.^	^.^
0.15 (−0.55; 0.86)	0.06 (−0.53; 0.66)	0.25 (−0.66; 1.17)	ST	0.23 (−0.87; 1.33)
0.79 (0.18; 1.40)	0.70 (0.28; 1.12)	0.89 (0.06; 1.72)	0.64 (0.02; 1.25)	CG

The upper triangle reports estimates from direct head-to-head trials. A “.” denotes the absence of such direct evidence. The lower triangle presents frequentist network meta-analysis results, integrating both direct and indirect comparisons across the treatment network. The light green area in the upper triangle displays estimates from direct head-to-head trials, with “.” indicating the absence of direct evidence. The yellow area ranks the standardized mean differences for various interventions, and the orange area in the lower triangle shows the network meta-analysis results, combining both direct and indirect comparisons.

### Quality of life

Among the 11 studies (*N* = 876), Mind–body training (MT) most effectively boosted quality of life (SMD = 1.02, 95% CI: −0.43 to 2.48), with a *p*-value of 0.89 (Figure 2C). Although the 95% confidence interval included zero, suggesting that the effect may not be statistically significant and indicating the need for further statistical validation, MT still outperformed other interventions in overall effectiveness. Mixed physical activity (MP) and strength training (ST) had comparatively weaker effects in enhancing quality of life, with SMDs of 0.32 (95% CI: −0.16 to 0.81) and 0.07 (95% CI: −0.61 to 0.75), and *p*-value of 0.46 and 0.27, respectively (Figure 4C). The control group (CG) had the lowest effectiveness (SMD = 0.00, *p*-value 0.02). Table 3 highlights MT with the highest *p*-value for QoL improvement (*p*-value = 0.89), making it the preferred intervention choice.

**Table 3:** League table of standardized mean differences for QoL interventions.

MT	^.^	0.95 (−0.34; 2.24)	^.^
0.70 (−0.79; 2.20)	MP	0.14 (−1.19; 1.48)	0.34 (−0.17; 0.85)
0.95 (−0.34; 2.24)	0.25 (−0.51; 1.01)	ST	0.03 (−0.74; 0.81)
1.02 (−0.43; 2.48)	0.32 (−0.16; 0.81)	0.07 (−0.61; 0.75)	CG

The upper triangle reports estimates from direct head-to-head trials. A ‘.’ denotes the absence of such direct evidence. The lower triangle presents frequentist network meta-analysis results, integrating both direct and indirect comparisons across the treatment network. The light green area in the upper triangle reports estimates from direct head-to-head trials, with “.” denoting the absence of such direct evidence. The yellow area ranks the standardized mean differences for various interventions, while the orange area in the lower triangle presents the network meta-analysis results, combining direct and indirect comparisons.

### Heterogeneity and consistency analysis

**Figure 5:**
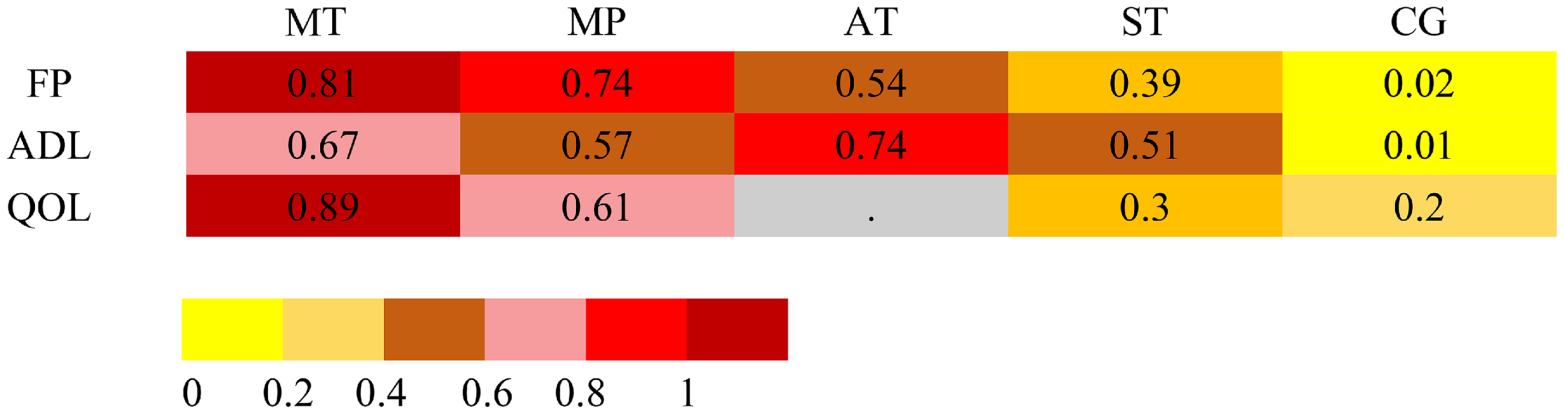
Intervention rankings across frailty, ADLs, and QoL outcomes based on frequentist network meta-analysis. Rankings are derived from P-scores, which estimate the probability that one treatment is superior to others by synthesizing point estimates and standard errors across the network. A dot (“.”) denotes that the corresponding intervention was not evaluated for that specific outcome. Abbreviations: MT, mind–body training; MP, mixed physical activity; AT, aerobic training; ST, strength training; CG, control group.

The network meta-analysis assessed heterogeneity using the I^2^ and τ^2^ statistics and evaluated consistency by point splitting methods. The analysis indicated low heterogeneity across all health metrics (I^2^ = 45.2 to 65.5%, τ^2^ = 0.01 to 0.29), suggesting stable and consistent results from the included studies. No significant inconsistency hotspots were detected (Appendices 7, 8; Figure 5), suggesting that there was no systematic bias between direct and indirect evidence. In addition, the robustness of the results was further verified by funnel plots (Appendix 10) and mesh plots (Figure 2), confirming the absence of significant publication bias (Figure 3; Appendix 6, Figures S1–S3). Collectively, these analyses further substantiate the reliability and validity of various physical activity interventions in enhancing health outcomes in older adults, offering a robust evidence-based basis for developing clinical intervention programs.

### Main findings

The network meta-analysis revealed differences between physical activity interventions in terms of their effectiveness in reducing frailty, enhancing activities of daily living, and improving quality of life in older adults. The results indicated that Mind–body training (e.g., Taiji, Baduanjin), exemplified by a traditional Chinese sports program that integrates gentle body movements with controlled breathing, were highly effective in improving the debilitating condition of the older adult and significantly enhancing their quality of life. In addition, aerobic training proved especially beneficial in improving activities of daily living, effectively enhancing their performance of ADLs independently. Mixed physical activity (e.g., combining strength training and flexibility training) demonstrated overall effectiveness across all health indicators. It is an effective strategy to improve the overall health of older adults. Therefore, the relative effectiveness of various physical activity interventions should be thoroughly considered in developing clinical intervention protocols, with a focus on mind–body training to enhance frailty and quality of life. Optimal combinations of physical activities tailored to individual differences and specific health needs will further enhance comprehensive health improvement in older adult individuals, providing a reliable evidence-based foundation for managing frailty.

## Discussion

This network meta-analysis compared the effects of various physical activities on frailty, the ability to perform activities of daily living, and quality of life among older adults. The findings revealed that there were significant disparities in how different physical activities influence the enhancement of these indicators: Mind–body training (MT) yielded the most notable improvements in frailty and quality of life; aerobic training was the most effective in enhancing the ability to perform activities of daily living; and mixed physical activity (MP) showed considerable benefits in alleviating frailty and improving ADLs performance, albeit with a lesser effect on enhancing quality of life. Strength training (ST) demonstrated a relatively more modest effect across the indicators. The analysis also indicated that variables such as the frequency and duration of interventions did not significantly moderate the outcomes of physical activity. These results provide various clinical and intervention strategies for managing frailty in older adults.

In line with previous research, physical activity remains foundational in mitigating frailty by enhancing musculoskeletal function and lowering risk profiles in older adults (37, 38). Mind–body practices such as Tai Chi and Baduanjin, known for promoting balance and proprioception, have shown particular efficacy—findings that our analysis corroborates (39). Similarly, aerobic and resistance-based regimens have been widely reported to improve muscle strength and endurance (40). Meanwhile, mixed exercise interventions, which integrate aerobic, strength, and flexibility components, have demonstrated comprehensive health benefits, including improved cardiorespiratory fitness, coordination, muscular endurance, and reduced frailty severity (41).

Previous studies have often explored pharmacological interventions (e.g., hormone therapy) to mitigate debilitating symptoms by enhancing muscle mass and strength; however, their clinical applicability remains limited due to adverse effects such as cardiovascular complications and sleep apnea exacerbation (42). Consequently, physical activity has emerged as a superior alternative, presenting fewer risks and more substantial long-term benefits in combating age-related neuromuscular impairments, thereby promoting physical function and QoL among older adults (43, 44). In our analysis, Mind–body training significantly improved frailty (SMD = −0.71, 95% CI: −1.22 ~ −0.21) and QoL (SMD = 1.02, 95% CI: −0.43 ~ 2.48), surpassing other physical activity forms. This aligns with evidence suggesting Mind–body modalities not only improve muscle strength but also enhance neurological function by activating motor cortical areas and facilitating central nervous system remodeling (45–51).

Aerobic training also yielded notable gains in mobility (SMD = 0.89, 95% CI: 0.06 ~ 1.72), aligning with earlier findings regarding its neuroprotective effects, such as the preservation of gray and white matter volume (16, 52) and the enhancement of neuromuscular efficiency through mitochondrial biogenesis and improved antioxidant defense mechanisms (53). These physiological adaptations collectively improve daily functional capacity and lower the risk of falls, outcomes that are particularly relevant in geriatric populations. Ma et al. similarly demonstrated that structured, progressively intensified aerobic programs significantly improved ADL performance in older adults, especially when supervision was maintained throughout the intervention (54). Together, these findings corroborate the evidence base for aerobic training as a core component in frailty interventions and validate the robustness of our network meta-analytic results (55–57).

Despite abundant evidence supporting physical activity interventions, existing research frequently remains fragmented, predominantly examining single-exercise modalities or being constrained by methodological inconsistencies and varied participant profiles (58). Additionally, studies often overlook comprehensive comparative assessments of various exercise modalities, restricting a nuanced understanding of their differential benefits (59). By adopting a rigorous network meta-analysis approach, this study systematically addressed these methodological gaps, distinctly clarifying the relative strengths and clinical implications of diverse physical activity modalities. Such comprehensive comparative analyses provide robust and actionable evidence to facilitate individualized and targeted frailty intervention strategies.

Collectively, these results emphasize the necessity for clinicians and healthcare providers to strategically select and integrate physical activity interventions based on patient-specific health conditions and functional needs, ultimately enhancing the overall efficacy and sustainability of frailty management strategies among aging populations.

### Clinical significance

The findings of this comprehensive network meta-analysis provide essential insights into clinical strategies for managing frailty among the growing global population of older adults. The distinct efficacy patterns observed across various physical activity interventions offer clinicians clear guidance for personalized patient care. Specifically, Mind–body training emerged as most effective in alleviating frailty and enhancing overall quality of life, making it particularly suitable for severely frail individuals with restricted mobility (60). Meanwhile, aerobic training demonstrated notable advantages in improving activities of daily living (ADLs), thus playing a pivotal role in preserving functional independence—an essential goal in geriatric health management given its direct impact on life quality and reduced healthcare burden (61, 62).

Furthermore, the identified flexibility in optimal intervention duration (averaging approximately 18 weeks), frequency (2–7 sessions weekly), and session length (20–80 min) underscores the practicality and feasibility of customizing physical activity regimens based on individual patient needs and contextual constraints. This personalized and adaptable approach not only enhances patient adherence and intervention effectiveness but also aligns well with current recommendations advocating individualized geriatric care. Ultimately, this study equips clinicians and healthcare policymakers with robust, evidence-based strategies for effectively addressing frailty, thereby significantly contributing to healthier aging trajectories and improved quality of life among older adults.

## Strengths

This study possesses several notable strengths. First, a comprehensive and systematic search strategy was employed across major international databases, encompassing both published and unpublished randomized controlled trials (RCTs), without restrictions on language or publication date. This exhaustive approach enhanced the generalizability and validity of the findings. Second, the use of a network meta-analysis (NMA) framework allowed for the simultaneous comparison of multiple physical activity modalities, integrating both direct and indirect evidence. This methodological advantage improved the precision and interpretability of effect estimates. Third, the inclusion of 35 RCTs comprising 2,905 participants strengthened the statistical robustness of the analysis. Finally, by evaluating a broad range of exercise types—Mind–body training, aerobic training, strength training, and mixed modalities—this study offers clinicians and researchers valuable guidance on tailoring interventions to manage frailty in older adults.

## Limitations

Despite its methodological strengths, several limitations warrant consideration. First, substantial heterogeneity existed in the intensity, frequency, and duration of interventions, which may affect result comparability and external validity. Second, many trials lacked detailed reporting of confounding variables such as diet, psychological status, and medication use, potentially introducing residual bias. Third, the validity of network meta-analysis depends on the assumptions of transitivity and consistency; although no major inconsistencies were detected, unmeasured effect modifiers may have biased the estimates. Fourth, the reliance on summary-level data limited the ability to conduct subgroup analyses and examine individual-level heterogeneity. Finally, although no language restrictions were applied, some relevant studies, particularly those not indexed in the searched databases, may have been inadvertently missed, leading to potential retrieval or publication bias. These limitations reflect common challenges in evidence synthesis and underscore the need for more standardized reporting and harmonized methodologies in future research on geriatric exercise interventions.

## Conclusion

This NMA suggests notable distinctions between various physical activities in terms of frailty, the ability to perform activities of daily living, and quality of life in older adults. Specifically, interventions that combined Mind–body training emerged as the most beneficial for reducing frailty, whereas mixed activity significantly improved overall fitness and ADLs. Aerobic training was particularly effective in improving the ability to perform activities of daily living. Consequently, it is recommended that clinicians tailor physical activity combinations to individual patient needs to optimize quality of life and autonomy in the older adult population. The long-term effects of different physical activities should continue to be explored in the future, and their efficacy across different cultural and health contexts should be assessed through rigorous trials. The development of personalized and varied interventions for frailty will become increasingly important as aging populations, not only to help improve the health of older adults but also to alleviate public health pressure.

## Data Availability

The raw data supporting the conclusions of this article will be made available by the authors, without undue reservation.
