# Supplementary material for: Mind–body training outperforms other physical activities in reducing frailty and enhancing quality of life in older adults: a network meta-analysis
Source: Front Public Health. 2025 Jul 14;13:1578791. doi: 10.3389/fpubh.2025.1578791 (PMC12301383; doi:10.3389/fpubh.2025.1578791)
Supplement: Supplementary file 1 [file Table_1.docx]

**Mind-Body Training Outperforms Other Physical Activities in Reducing Frailty and Enhancing Quality of Life in Older Adults: A Network Meta-Analysis**

**LIU guangwen^1^, GE renkai^1*^, ZHU huiling^1^**

1. School of Physical Education and Health, East China Jiaotong University, Nanchang, China.

***Corresponding Author**

GE Renkai

rkge@ecjtu.edu.cn

**Supplemental files**

**Catalog**

[Appendix 1: PRISMA NMA Checklist of Items to Include When Reporting A Systematic Review Involving a Network Meta-analysis 1](#_Toc142306353)

Appendix 2. Searching strategies........................................................................................................................................................11

Appendix3. A List of Pre-specified Eligible Instruments for Measuring Frailty, Daily Activities, and Quality of Life...................13

[Appendix 4. Coding guide and description of frailty interventions.](#_Toc16928)...................................................................................................14

[Appendix 5. Main characteristics of included randomized controlled trials .](#_Toc11609).....................................................................................15

Appendix 6.Risk of bias......................................................................................................................................................................21

Appendix 7. Forest plot of outcomes..................................................................................................................................................23

Appendix 8. Hot spots of inconsistency..............................................................................................................................................24

Appendix 9. Percentage contribution matrices...................................................................................................................................26

Appendix 10. Network meta-analysis funnel plots ............................................................................................................................28

[Appendix 11. List of included studies..](#_Toc8198)...............................................................................................................................................29

| **Section/Topic** | **Item #** | **Checklist Item** | **Reported on Page #** |
| --- | --- | --- | --- |
| **TITLE** |  |  |  |
| Title | 1 | Identify the report as a systematic review *incorporating a network meta-analysis (or related form of meta-analysis).* | **Page. 1-2**  **Title page** |
|  |  |  |  |
| **ABSTRACT** |  |  |  |
| Structured summary | 2 | Provide a structured summary including, as applicable:  **Background:** main objectives  **Methods:** data sources; study eligibility criteria, participants, and interventions; study appraisal; and *synthesis methods, such as network meta-analysis.*  **Results:** number of studies and participants identified; summary estimates with corresponding confidence/credible intervals; *treatment rankings may also be discussed. Authors may choose to summarize pairwise comparisons against a chosen treatment included in their analyses for brevity.*  **Discussion/Conclusions:** limitations; conclusions and implications of findings.  **Other:** primary source of funding; systematic review registration number with registry name. | **Page. 3** |
|  |  |  |  |
| **INTRODUCTION** |  |  |  |
| Rationale | 3 | Describe the rationale for the review in the context of what is already known*, including mention of why a network meta-analysis has been conducted.* | **Page. 4-5** |
| Objectives | 4 | Provide an explicit statement of questions being addressed, with reference to participants, interventions, comparisons, outcomes, and study design (PICOS). | **Page. 5-6** |
|  |  |  |  |
| **METHODS** |  |  |  |
| Protocol and registration | 5 | Indicate whether a review protocol exists and if and where it can be accessed (e.g., Web address); and, if available, provide registration information, including registration number. | **NA** |
| Eligibility criteria | 6 | Specify study characteristics (e.g., PICOS, length of follow-up) and report characteristics (e.g., years considered, language, publication status) used as criteria for eligibility, giving rationale. *Clearly describe eligible treatments included in the treatment network, and note whether any have been clustered or merged into the same node (with justification).* | **Page. 7** |
| Information sources | 7 | Describe all information sources (e.g., databases with dates of coverage, contact with study authors to identify additional studies) in the search and date last searched. | **Page. 7** |
| Search | 8 | Present full electronic search strategy for at least one database, including any limits used, such that it could be repeated. | **Appendix 2** |
| Study selection | 9 | State the process for selecting studies (i.e., screening, eligibility, included in systematic review, and, if applicable, included in the meta-analysis). | **Page. 7** |
| Data collection process | 10 | Describe method of data extraction from reports (e.g., piloted forms, independently, in duplicate) and any processes for obtaining and confirming data from investigators. | **Page. 8** |
| Data items | 11 | List and define all variables for which data were sought (e.g., PICOS, funding sources) and any assumptions and simplifications made. | **Page. 7-8** |
| Geometry of the network | **S1** | Describe methods used to explore the geometry of the treatment network under study and potential biases related to it. This should include how the evidence base has been graphically summarized for presentation, and what characteristics were compiled and used to describe the evidence base to readers. | **Page. 8** |
| Risk of bias within individual studies | 12 | Describe methods used for assessing risk of bias of individual studies (including specification of whether this was done at the study or outcome level), and how this information is to be used in any data synthesis. | **Page. 8** |
| Summary measures | 13 | State the principal summary measures (e.g., risk ratio, difference in means). *Also describe the use of additional summary measures assessed, such as treatment rankings and surface under the cumulative ranking curve (SUCRA) values, as well as modified approaches used to present summary findings from meta-analyses.* | **Page. 9** |
| Planned methods of analysis | 14 | Describe the methods of handling data and combining results of studies for each network meta-analysis. This should include, but not be limited to:   - *Handling of multi-arm trials;* - *Selection of variance structure;* - *Selection of prior distributions in Bayesian analyses; and* - *Assessment of model fit.* | **Page.7-9** |
| **Assessment of Inconsistency** | **S2** | Describe the statistical methods used to evaluate the agreement of direct and indirect evidence in the treatment network(s) studied. Describe efforts taken to address its presence when found. | **Page. 8-9** |
| Risk of bias across studies | 15 | Specify any assessment of risk of bias that may affect the cumulative evidence (e.g., publication bias, selective reporting within studies). | **Page. 9** |
| Additional analyses | 16 | Describe methods of additional analyses if done, indicating which were pre-specified. This may include, but not be limited to, the following:   - Sensitivity or subgroup analyses; - Meta-regression analyses; - *Alternative formulations of the treatment network; and* - *Use of alternative prior distributions for Bayesian analyses (if applicable).* | **Page. 9-10** |
|  |  |  |  |
| **RESULTS†** |  |  |  |
| Study selection | 17 | Give numbers of studies screened, assessed for eligibility, and included in the review, with reasons for exclusions at each stage, ideally with a flow diagram. | **Page.10**  **Figure 1** |
| **Presentation of network structure** | **S3** | Provide a network graph of the included studies to enable visualization of the geometry of the treatment network. | **Figure 2** |
| **Summary of network geometry** | **S4** | Provide a brief overview of characteristics of the treatment network. This may include commentary on the abundance of trials and randomized patients for the different interventions and pairwise comparisons in the network, gaps of evidence in the treatment network, and potential biases reflected by the network structure. | **Page.11**  **Figure3** |
| Study characteristics | 18 | For each study, present characteristics for which data were extracted (e.g., study size, PICOS, follow-up period) and provide the citations. | **Page.11**  **Appendix 5** |
| Risk of bias within studies | 19 | Present data on risk of bias of each study and, if available, any outcome level assessment. | **Page.11-12**  **Appendix 6** |
| Results of individual studies | 20 | For all outcomes considered (benefits or harms), present, for each study: 1) simple summary data for each intervention group, and 2) effect estimates and confidence intervals. *Modified approaches may be needed to deal with information from larger networks.* | **Page12**  **Appendix 7-10** |
| Synthesis of results | 21 | Present results of each meta-analysis done, including confidence/credible intervals. *In larger networks, authors may focus on comparisons versus a particular comparator (e.g. placebo or standard care), with full findings presented in an appendix. League tables and forest plots may be considered to summarize pairwise comparisons.* If additional summary measures were explored (such as treatment rankings), these should also be presented. | **Page.11**  **Appendix 7-10**  **Figure 3-4，**  **Table 4** |
| **Exploration for inconsistency** | **S5** | Describe results from investigations of inconsistency. This may include such information as measures of model fit to compare consistency and inconsistency models, *P* values from statistical tests, or summary of inconsistency estimates from different parts of the treatment network. | **Page.12-13**  **Figure5** |
| Risk of bias across studies | 22 | Present results of any assessment of risk of bias across studies for the evidence base being studied. | **Page.13**  **Appendix 6** |
| Results of additional analyses | 23 | Give results of additional analyses, if done (e.g., sensitivity or subgroup analyses, meta-regression analyses*, alternative network geometries studied, alternative choice of prior distributions for Bayesian analyses,* and so forth). | **Page14** |
|  |  |  |  |
| **DISCUSSION** |  |  |  |
| Summary of evidence | 24 | Summarize the main findings, including the strength of evidence for each main outcome; consider their relevance to key groups (e.g., healthcare providers, users, and policy-makers). | **Page.15-16** |
| Limitations | 25 | Discuss limitations at study and outcome level (e.g., risk of bias), and at review level (e.g., incomplete retrieval of identified research, reporting bias). *Comment on the validity of the assumptions, such as transitivity and consistency. Comment on any concerns regarding network geometry (e.g., avoidance of certain comparisons).* | **Page.17** |
| Conclusions | 26 | Provide a general interpretation of the results in the context of other evidence, and implications for future research. | **Page.18-19** |
|  |  |  |  |
| **FUNDING** |  |  |  |
| Funding | 27 | This study was funded by National Natural Science Foundation of China (NSFC) and the Postgraduate Innovation Special Fund Project (YC2024-S443). |  |

| **Section/Topic** | **Item #** | **Checklist Item** | **Reported on Page #** |
| --- | --- | --- | --- |
| **TITLE** |  |  |  |
| Title | 1 | Identify the report as a systematic review *incorporating a network meta-analysis (or related form of meta-analysis).* | **Page. 1**  **Title page** |
|  |  |  |  |
| **ABSTRACT** |  |  |  |
| Structured summary | 2 | Provide a structured summary including, as applicable:  **Background:** main objectives  **Methods:** data sources; study eligibility criteria, participants, and interventions; study appraisal; and *synthesis methods, such as network meta-analysis.*  **Results:** number of studies and participants identified; summary estimates with corresponding confidence/credible intervals; *treatment rankings may also be discussed. Authors may choose to summarize pairwise comparisons against a chosen treatment included in their analyses for brevity.*  **Discussion/Conclusions:** limitations; conclusions and implications of findings.  **Other:** primary source of funding; systematic review registration number with registry name. | **Page. 1-2** |
|  |  |  |  |
| **INTRODUCTION** |  |  |  |
| Rationale | 3 | Describe the rationale for the review in the context of what is already known*, including mention of why a network meta-analysis has been conducted.* | **Page. 2** |
| Objectives | 4 | Provide an explicit statement of questions being addressed, with reference to participants, interventions, comparisons, outcomes, and study design (PICOS). | **Page. 2-3** |
|  |  |  |  |
| **METHODS** |  |  |  |
| Protocol and registration | 5 | Indicate whether a review protocol exists and if and where it can be accessed (e.g., Web address); and, if available, provide registration information, including registration number. | **NA** |
| Eligibility criteria | 6 | Specify study characteristics (e.g., PICOS, length of follow-up) and report characteristics (e.g., years considered, language, publication status) used as criteria for eligibility, giving rationale. *Clearly describe eligible treatments included in the treatment network, and note whether any have been clustered or merged into the same node (with justification).* | **Page. 3-4** |
| Information sources | 7 | Describe all information sources (e.g., databases with dates of coverage, contact with study authors to identify additional studies) in the search and date last searched. | **Page. 3** |
| Search | 8 | Present full electronic search strategy for at least one database, including any limits used, such that it could be repeated. | **Appendix 2** |
| Study selection | 9 | State the process for selecting studies (i.e., screening, eligibility, included in systematic review, and, if applicable, included in the meta-analysis). | **Page. 3** |
| Data collection process | 10 | Describe method of data extraction from reports (e.g., piloted forms, independently, in duplicate) and any processes for obtaining and confirming data from investigators. | **Page. 3** |
| Data items | 11 | List and define all variables for which data were sought (e.g., PICOS, funding sources) and any assumptions and simplifications made. | **Page. 3-4** |
| Geometry of the network | **S1** | Describe methods used to explore the geometry of the treatment network under study and potential biases related to it. This should include how the evidence base has been graphically summarized for presentation, and what characteristics were compiled and used to describe the evidence base to readers. | **Page. 4** |
| Risk of bias within individual studies | 12 | Describe methods used for assessing risk of bias of individual studies (including specification of whether this was done at the study or outcome level), and how this information is to be used in any data synthesis. | **Page. 3-4** |
| Summary measures | 13 | State the principal summary measures (e.g., risk ratio, difference in means). *Also describe the use of additional summary measures assessed, such as treatment rankings and surface under the cumulative ranking curve (SUCRA) values, as well as modified approaches used to present summary findings from meta-analyses.* | **Page. 3-4** |
| Planned methods of analysis | 14 | Describe the methods of handling data and combining results of studies for each network meta-analysis. This should include, but not be limited to:   - *Handling of multi-arm trials;* - *Selection of variance structure;* - *Selection of prior distributions in Bayesian analyses; and* - *Assessment of model fit.* | **Page. 4** |
| **Assessment of Inconsistency** | **S2** | Describe the statistical methods used to evaluate the agreement of direct and indirect evidence in the treatment network(s) studied. Describe efforts taken to address its presence when found. | **Page. 3-4** |
| Risk of bias across studies | 15 | Specify any assessment of risk of bias that may affect the cumulative evidence (e.g., publication bias, selective reporting within studies). | **Page. 4** |
| Additional analyses | 16 | Describe methods of additional analyses if done, indicating which were pre-specified. This may include, but not be limited to, the following:   - Sensitivity or subgroup analyses; - Meta-regression analyses; - *Alternative formulations of the treatment network; and* - *Use of alternative prior distributions for Bayesian analyses (if applicable).* | **Page. 3-4** |
| **RESULTS†** |  |  |  |
| Study selection | 17 | Give numbers of studies screened, assessed for eligibility, and included in the review, with reasons for exclusions at each stage, ideally with a flow diagram. | **Page.4**  **Figure 1** |
| **Presentation of network structure** | **S3** | Provide a network graph of the included studies to enable visualization of the geometry of the treatment network. | **Figure 2** |
| **Summary of network geometry** | **S4** | Provide a brief overview of characteristics of the treatment network. This may include commentary on the abundance of trials and randomized patients for the different interventions and pairwise comparisons in the network, gaps of evidence in the treatment network, and potential biases reflected by the network structure. | **Page.5**  **Figure3** |
| Study characteristics | 18 | For each study, present characteristics for which data were extracted (e.g., study size, PICOS, follow-up period) and provide the citations. | **Page.5**  **Appendix 5** |
| Risk of bias within studies | 19 | Present data on risk of bias of each study and, if available, any outcome level assessment. | **Page.5**  **Appendix 6** |
| Results of individual studies | 20 | For all outcomes considered (benefits or harms), present, for each study: 1) simple summary data for each intervention group, and 2) effect estimates and confidence intervals. *Modified approaches may be needed to deal with information from larger networks.* | **Page.5-6**  **Appendix 7-10** |
| Synthesis of results | 21 | Present results of each meta-analysis done, including confidence/credible intervals. *In larger networks, authors may focus on comparisons versus a particular comparator (e.g. placebo or standard care), with full findings presented in an appendix. League tables and forest plots may be considered to summarize pairwise comparisons.* If additional summary measures were explored (such as treatment rankings), these should also be presented. | **Page.5-6**  **Appendix 7-10**  **Figure 3-4，**  **Table 3-4** |
| **Exploration for inconsistency** | **S5** | Describe results from investigations of inconsistency. This may include such information as measures of model fit to compare consistency and inconsistency models, *P* values from statistical tests, or summary of inconsistency estimates from different parts of the treatment network. | **Page.6**  **Figure5** |
| Risk of bias across studies | 22 | Present results of any assessment of risk of bias across studies for the evidence base being studied. | **Page.5**  **Appendix 6** |
| Results of additional analyses | 23 | Give results of additional analyses, if done (e.g., sensitivity or subgroup analyses, meta-regression analyses*, alternative network geometries studied, alternative choice of prior distributions for Bayesian analyses,* and so forth). | **Page5-6** |
|  |  |  |  |
| **DISCUSSION** |  |  |  |
| Summary of evidence | 24 | Summarize the main findings, including the strength of evidence for each main outcome; consider their relevance to key groups (e.g., healthcare providers, users, and policy-makers). | **Page.6-7** |
| Limitations | 25 | Discuss limitations at study and outcome level (e.g., risk of bias), and at review level (e.g., incomplete retrieval of identified research, reporting bias). *Comment on the validity of the assumptions, such as transitivity and consistency. Comment on any concerns regarding network geometry (e.g., avoidance of certain comparisons).* | **Page.7** |
| Conclusions | 26 | Provide a general interpretation of the results in the context of other evidence, and implications for future research. | **Page.8** |
|  |  |  |  |
| **FUNDING** |  |  |  |
| Funding | 27 | This study was funded by National Natural Science Foundation of China (NSFC) and the Postgraduate Innovation Special Fund Project (YC2024-S443). |  |

**Appendix 1: PRISMA NMA Checklist of Items to Include When Reporting A Systematic Review Involving a Network Meta-analysis**

PICOS = population, intervention, comparators, outcomes, study design.

* Text in italics indicates wording specific to reporting of network meta-analyses that has been added to guidance from the PRISMA statement.

† Authors may wish to plan for use of appendices to present all relevant information in full detail for items in this section.

# Appendix 2. Searching strategies.

| **Pubmed(searched on August 28, 2024)** | | |
| --- | --- | --- |
| #1 | Exercise(Mesh item) | 260605 |
| #2 | physical activity*[Title/Abstract] OR Activities”[Title/Abstract] OR “physical activities’=Title/Abstract]OR“Exercises”[Title/Abstract] OR“physical exercise”[Title/Abstract]OR “physical exercises [Title/Abstract]OR “acute exercise’「Title/Abstract OR “aerobic exercise"[Title/Abstract\|OR “aerobic exercises"Title/Abstract]OR“exercise training’「Title/Abstract]OR “exercise training s’ Title / Abstract OR “tai chi’「Title/Abstract OR “Yoga"[Title/Abstract]OR“PILATES [Title/Abstract]OR weightlifting"「Title/Abstract]OR “qi gong”[Title/Abstract]OR “baduaniin"Title/Abstract]OR 'swim [Title/Abstract])) | 1173718 |
| #3 | Or/1-2 | 1319514 |
| #4 | Aged(mesh) | 3556457 |
| #5 | ("Elderly"「Title/Abstract] OR "Aged"「Title/Abstract]) | 1037836 |
| #6 | Or/4-5 | 4163760 |
| #7 | ("Frailty"[MeSH Terms] | 832999 |
| #8 | ("Frailties"[Title/Abstract]OR "Frailness"[Title/Abstract OR "frailty syndrome"[Title/Abstract]OR"Debility"[Title/Abstract] OR "Debilities"[Title/Abstract]) | 206231 |
| #9 | or/7-8 | 13252 |
| **Embase (searched on August 24, 2024)** | | |
| #1 | Exercise Frail Elderly/ | 10929 |
| #2 | exercise frail elderly.ti,ab,kw. | 5354 |
| #3 | frail*.ti,ab,kw. | 41747 |
| #4 | frailty$.ti,ab,kw. | 27673 |
| #5 | or/1-4 | 45106 |
| #6 | random:.tw. | 1694759 |
| #7 | limit 12 to yr="2001-Current" | 506 |
| **Web of Science (searched on August 24, 2024)** | | |
| #1 | TI='Frail' 'Exercise’ ‘Aged’ | [5,013](https://www.webofscience.com/wos/alldb/summary/b2aac444-ccab-4cda-94f8-808647ac8c2b-05d95a4d/relevance/1) |
| #2 | AB='Frail' 'Exercise’‘Aged’ | [15,409](https://www.webofscience.com/wos/alldb/summary/9c2e6cd2-d4de-4606-9e61-5ed26bef204d-05d95c93/relevance/1) |
| #3 | TI='Frailty' ‘Elderly’ | 12,496 |
| #4 | AB='Frailty' ‘Elderly’ | 18,976 |
| #5 | (((#4) OR #3) OR #2) OR #1 | 35,732 |
| #7 | 2001-01-01 to 2024-08-28 | 131 |
| **Cochrane Central Register of Controlled Trials (CENTRAL) (searched on August 24, 2024)** | | |
| #1 | MeSH descriptor: (Exercise) explode all trees | 495 |
| #2 | 'frail elderly'ti,ab,kw. | 431 |
| #3 | 'frail*' ti,ab,kw. | 621 |
| #4 | 'frailty$' ti,ab,kw. | 512 |
| #5 | (((#4) OR #3) OR #2) OR #1 | 255 |
| #6 | limit 5 to yr="2001-Current" | 492 |
| **China National Knowledge Infrastructure (CNKI) (Chinese) (searched on August 28, 2024)** | | |
|  | (TI='Frailty' OR AB='Frailties' OR TI'Frailness' ORAB=' Debility’)(TI='Exercise' OR AB='Physical' ORTI='Activity' OR AB='Acute Exerccise)(TI'Aged' ORAB→'Elderly') AND(SU%=RCT' ORFT='RCT') from 1 January 2001 to 28August 2024 | 34 |

**Appendix 3. A List of Pre-specified Eligible Instruments for Measuring Frailty, Daily Activities, and Quality of Life.**

**1. Frailty Measurement Instruments:**

1. Frailty phenotype (FP)
2. Clinical Frailty Scale (CFS)
3. FRAIL Scale (FS)
4. Study of Osteoporotic Fractures frailty criteria (SOF)
5. Tilburg Frailty Indicator (TFI)
6. Short Physical Performance Battery (SPPB)
7. Frailty Index (FI)
8. Edmonton Frailty Scale (EFS)

**2. Daily Activities Measurement Instruments:**

1. Barthel Index (BI)
2. Activities of Daily Living (ADLs)
3. Instrumental Activities of Daily Living (IADL)

**3. Quality of Life Measurement Instruments:**

1. EuroQol-5 Dimensions (EQ-5D)
2. Short Form-36 Health Survey (SF-36)
3. Short Form-8 Health Survey (SF-8)
4. Short Form-12 Health Survey (SF-12)

**Appendix 4. Coding guide and description of frailty interventions.**

| **Intervention** | **Codes** | **Definitions of coding an experimental intervention under the corresponding component** |
| --- | --- | --- |
| Aerobic training | AT | Doing exercises with sufficient oxygen supply (such as walking, cycling, jogging, swimming, etc). |
| Strength training | ST | Strength training focuses on increasing muscle strength and endurance through resistance exercises that challenge the muscles to adapt and grow stronger. |
| Mixed physical training | MP | A combination of two or more exercise types. |
| Psychophysical training | PT | Exercises that integrate physical movement with mental focus and controlled breathing, such as Tai Chi, yoga, and Qigong, aim to improve both physical and psychological health. |
| Control group | CG | Inert interventions without any exercise, such as keeping daily waiting lists and counseling education. |

**Appendix 5. Main characteristics of included randomized controlled trials.**

| First author Year | Country/Region | Types of intervention | Mean age (SD) (year) | Male (%) | Outcomes | Detailed comparisons | Frequency and Duration |
| --- | --- | --- | --- | --- | --- | --- | --- |
| Zech 2012 | Germany | Mixed physical training | 77.8 (6.1) | 23 (71.1) | SPPB，  SF-LLFDI | MP: Balance, resistance training | 60 min/session, 2 sessions/week  12 weeks |
| Jose 2016 | Spain | Mixed physical training | 79.7 (3.6) | 51 (43.0) | FP, EFS，  EQ-5D | MP: Aerobic, resistance, stretching, balance, flexibility training | 65 min/session, 5 or 7 session/week  24 weeks |
| Trombetti 2018 | Switzerland | Mixed physical training | 78.7 (5.2) | 812 (33.0) | SOF | MP: Aerobic, resistance, balance, flexibility training | 53-55 min/session,  5 session/week  104 weeks |
| Guo 2019 | China | Mixed physical training | 82.5 (4.2) | 29 (45.0) | FP | MP:resistance, balance, flexibility, aerobic training | 36-60 min/session, 3-5 session/week  12 weeks |
| Sadjapong 2020 | Thailand | Mixed physical training | 76.7(1.15) | 32 (28.0) | FS | MP:aerobic training, resistance training,  and balance training | 60 min/session, 3 sessions/week  12 weeks |
| Fu 2020 | China | Mixed physical training | 68.8 (2.3) | 53(53.0) | TFI | MP:aerobic training, resistance training, flexibility exercises  and balance training | NR,  2-3 session/week  12 weeks |
| Jiang 2020 | China | Mixed physical training | 72.5 (7.0) | 45 (NR) | FP, FI  ADL | MP: Stretching, resistance, aerobic training | NR,  5 session/week  12 weeks |
| Tamuleviciute 2021 | Lithuania | Mixed physical training | 76.1 (6.6) | 60 (50.0) | SPPB | MP: Resistance, balance, aerobic training | 35-40 min/session,  3 session/week  12 weeks |
| Nagaia 2018 | Japan | Multicomponent intervention | 81.2(7.3) | 21 (14.0) | FP，  SF;LADL | RT: Resistance training | NR,  2 session/week  24 weeks |
| Ge 2020 | China | Mind-body exercise | 70.2 (5.4) | 32 (34.0) | FP | PT: Tai Chi | 60 min/session, 3 session/week  8 weeks |
| Yoon 2017 | Korea | Resistance training | 75.0(3.5) | 9 (NR) | SPPB | RT: Resistance training | 60 min/session, 2 session/week  12 weeks |
| Yoon 2018 | Korea | Resistance training | 73.8(4.4) | 22 (30.0) | FP，SPPB | RT: Resistance training | 60 min/session, 3 session/week  16 weeks |
| Chen 2020 | USA | Resistance training | 77.0 (5.2) | 33 (36.0) | FP | RT: Resistance training | 45-60 min/session,  3 session/week  8 weeks |
| Hsieh 2019 | China | Mixed physical training | 72.2 (5.7) | 79 (61.0) | FP，  SF-12 | MP: Resistan, flexibilit, balance training | 5-60 min/session, 3-7 session/week  26 weeks |
| Karssemeijer 2019 | Netherlands | Multicomponent intervention | 79.4 (6.7) | 38 (53.0) | FI, SPPB | AT: Aerobic training | 30-50min/session,  3 session/week  12 weeks |
| Amasene 2021 | Spain | Strength training | 81.2 (6.1) | 20 (50.0) | SPPB | ST:Strength training | 60 min/session,  2 session/week  12 weeks |
| Arrieta  2019 | Spain | Mixed physical training | 85.1 (7.6) | 57 (26.0) | TFI | MP | 55 min/session,  7 session/week  24 weeks |
| Barrachina-Igual 2021 | Spain | Strength training | 74.8 (5.8) | 23 (30.0) | SPPB | ST:High intensity Strength training | 65 min/session,  2 session/week  12 weeks |
| Caldo-Silva  2021 | Portugal | Mixed physical training | 86.7 (4.0) | 7 (NR) | FI | MP | 20 min/session,  2 session/week  16 weeks |
| Furtado  2020 | Portugal | Psychophysical training | 80.1 (8.2) | 21 (21.0) | PF | PT:Chair-based exercise programs | 45 min/session,  2 session/week  28 weeks |
| Karssemeijer 2019 | Netherlands | Aerobic trainin | 80.9 (6.1) | 38 (33.0) | SPPB | AT:Cycling | 40 min/session,  3 session/week  12 weeks |
| Liao 2019 | China | Mixed physical training | 79.6 (8.5) | 24 (50.0) | FS | MP | 60 min/session,  3 session/week  12 weeks |
| Liu 2022 | China | Mixed physical training | 80.8 (3.0) | 67 (24.0) | FS | MP | 55 min/session,  5 session/week  48 weeks |
| Meng 2020 | China | Mixed physical training | 76.5 (6.5) | 74 (46.0) | FS | MP | 80 min/session,  3 session/week  12 weeks |
| Rezola-Pardo 2019 | Spain | Mixed physical training | 85.3 (7.1) | 43 (34.0) | TFI，  QOL | MP | 45 min/session,  7 session/week  12 weeks |
| Swales 2022 | UK | Strength training | 85.8 (7.8) | 6 (35.0) | FP,  SPPB | ST:Strength training | 37.5 min/session,  3 session/week  6 weeks |
| Chen 2019 | China | Strength training | 77.0 (5.2) | 33 (42.0) | FP,SF | ST:Elastic belt training | 52.5 min/session,  3 session/week  8 weeks |
| Hou 2018 | China | Psychophysical training | 82.9 (4.4) | 36 (28.0) | FI,  SF-36;ADL | PT:Baduanjin | 45 min/session,  5 session/week  12 weeks |
| Huang 2022 | China | Mixed physical training | 73.7 (7.1) | 57 (67.0) | TFI,SPPB | MP | 80 min/session,  2 session/week  3 weeks |
| Liu 2015 | China | Aerobic trainin | 73.9 (8.6) | 120 (58.0) | FI,SF-36 | AT:Aerobic walking | 65 min/session,  5 session/week  48 weeks |
| Zhang 2021 | China | Mixed physical training | 67.8 (4.9) | 38 (0) | FP | MP:Baduanjin and Resistance training | 75 min/session,  5 session/week  12 weeks |
| Zheng 2020 | China | Mixed physical training | 80.1 (5.4) | 30 (13.0) | TFI,SPPB | MP | 50 min/session,  3 session/week  12 weeks |
| Zhang, Xiaohong 2023 | Netherlands | Psychophysical training | 64.8 (4.4) | 36 (36.0) | FI,SPPB | PT:Dance Intervention | 60 min/session,  5 session/week  16 weeks |
| Yi-Jia Lin | Taiwan,  China | Aerobic training | 79.1 (6.8) | 23 (26.0) | FP | AT:Interactive boxing-riding | 50 min/session,  3 session/week  12 weeks |
| Sheng-Hui Tuan | Taiwan,  China | Mixed physical training | 75.8 (6.4) | 30 (33.0) | SOF | MP:Sports games Resistance, Aerobics and Balance training | 30 min/session,  2 session/week  12 weeks |

Notes: A: number of participants analyzed; EFS: Edmonton Frailty Scale; FI: Frailty Index; FP: Frailty phenotype; FS: Frail Scale; NR: not reported; R: number of participants randomized; SD: standard deviation; SOF: Study of Osteoporotic Fractures frailty criteria; SPPB: Short Physical Performance Battery; TFI: Tilburg Frailty Indicator; UK: United Kingdom; USA: United States;SF-36:Short Form-36 Health Survey;EQ-5D:EuroQol-5 Dimensions;BI:Barthel Index;ADL:Activities of Daily LivingI;IADL:Instrumental Activities of Daily Living;

**Appendix 6:** **Risk of bias**

**6.1 Risk of bias summary**

**
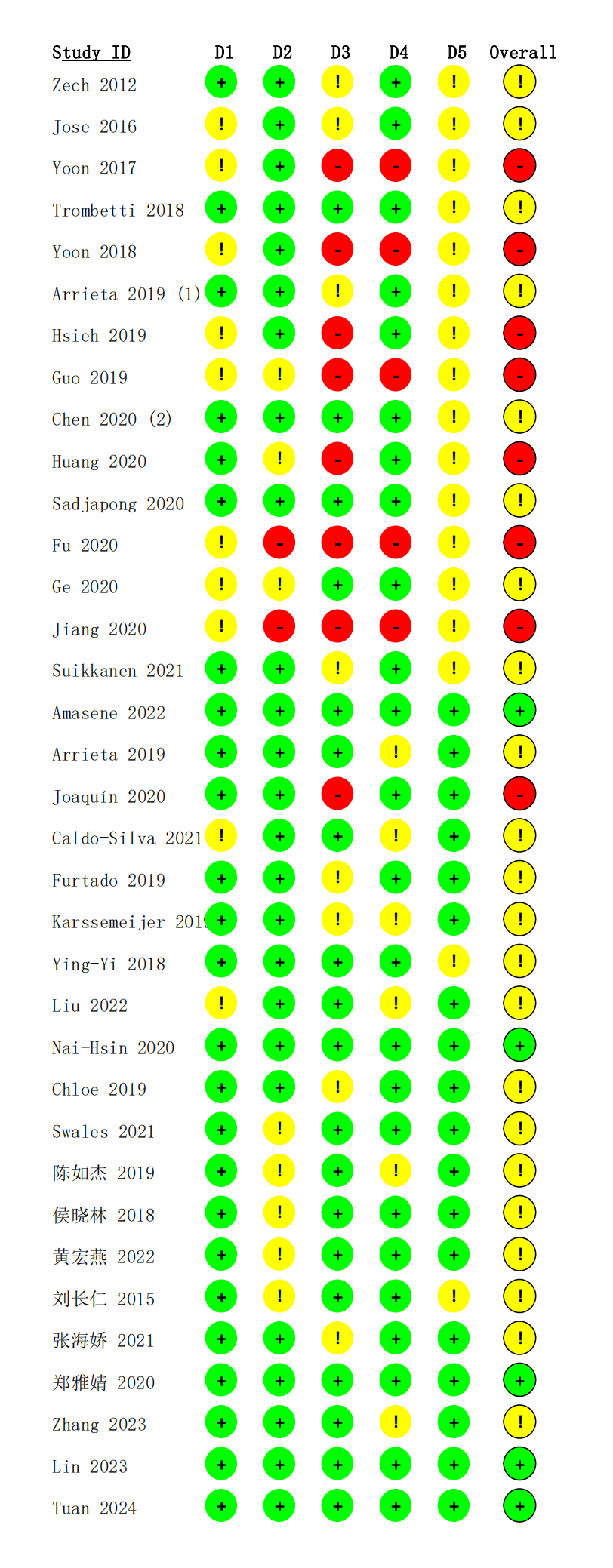
**

**Figure S1** Risk of bias summary for frailty.

**
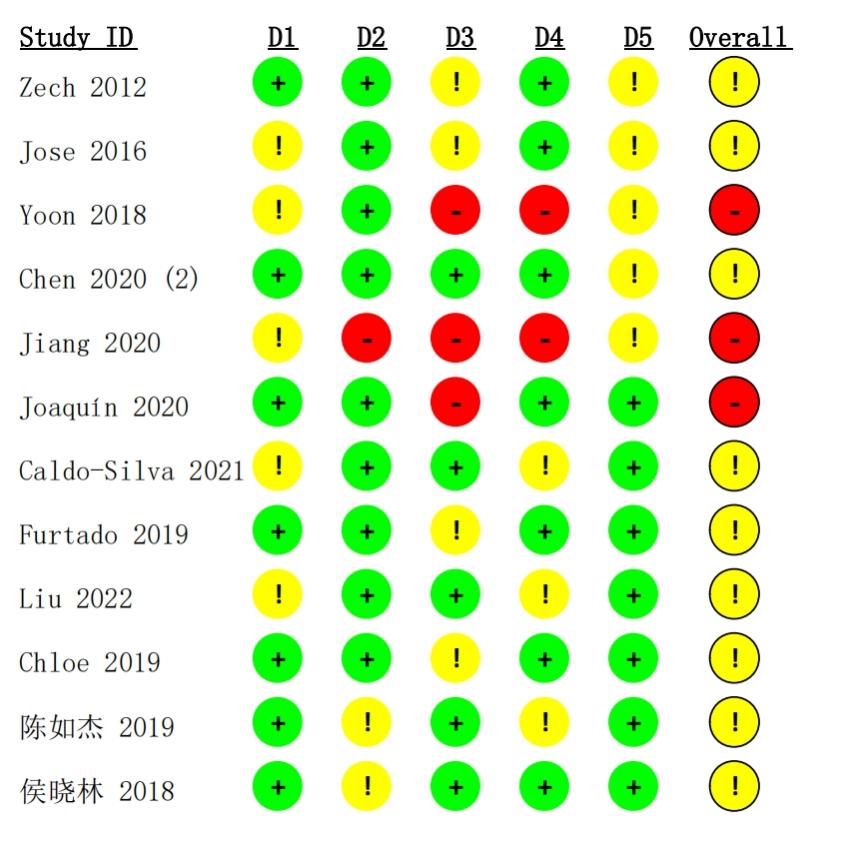
**

**Figure S2** Risk of bias summary for activities of daily living.

**
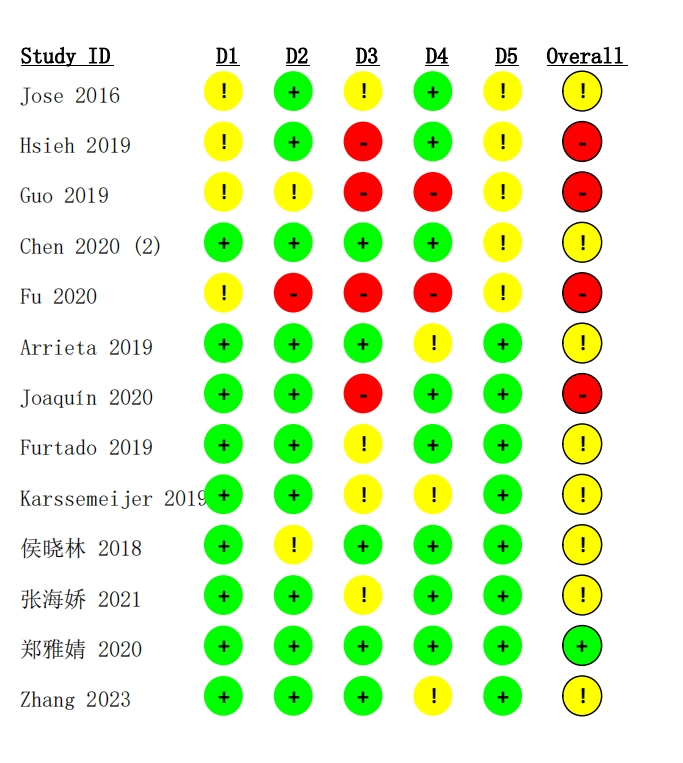
**

**Fi****gure S3** Risk of bias summary for quality of life.

**Appendix 7: Forest plot of outcomes**

**
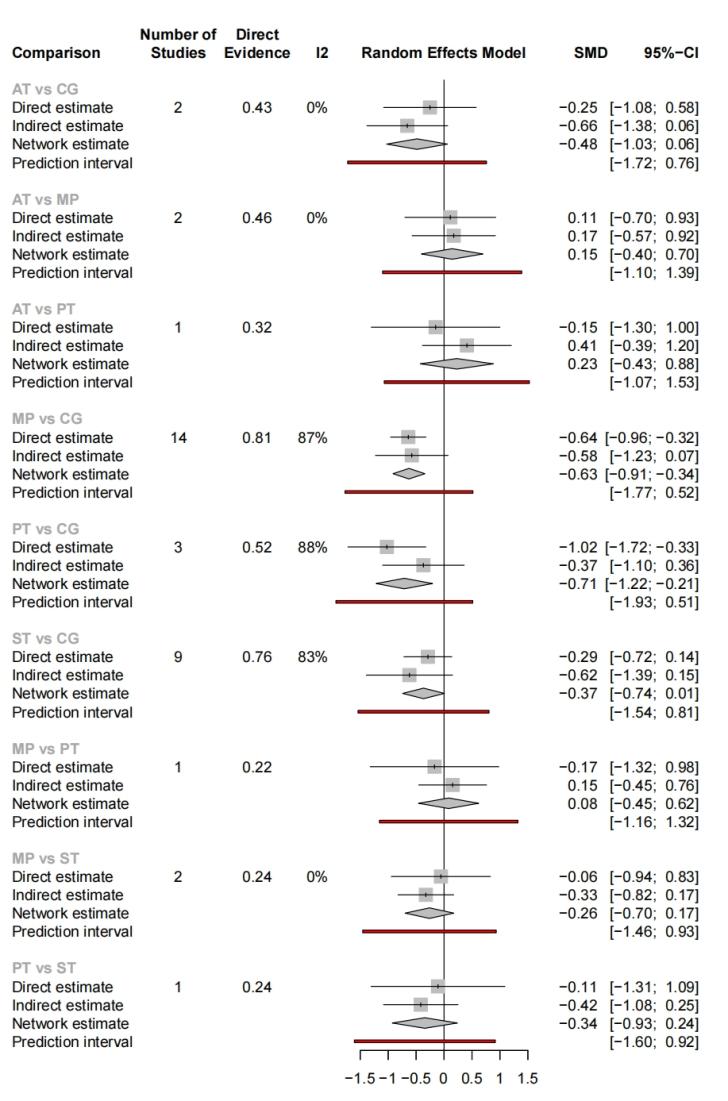

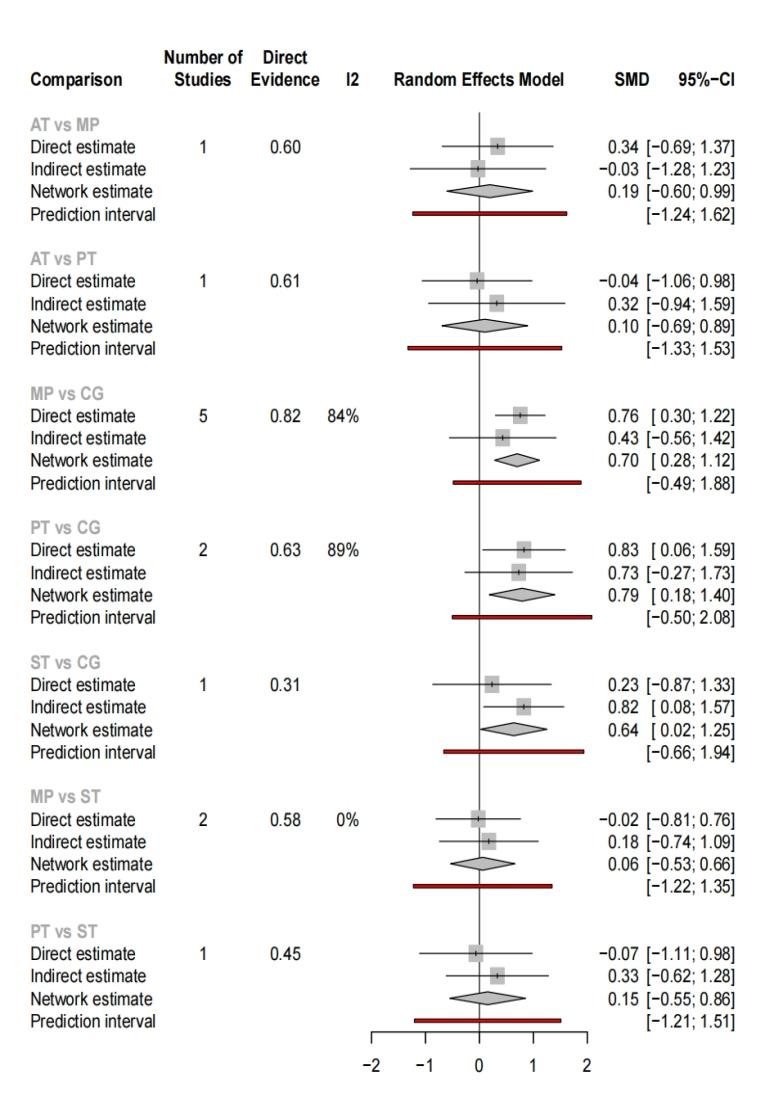

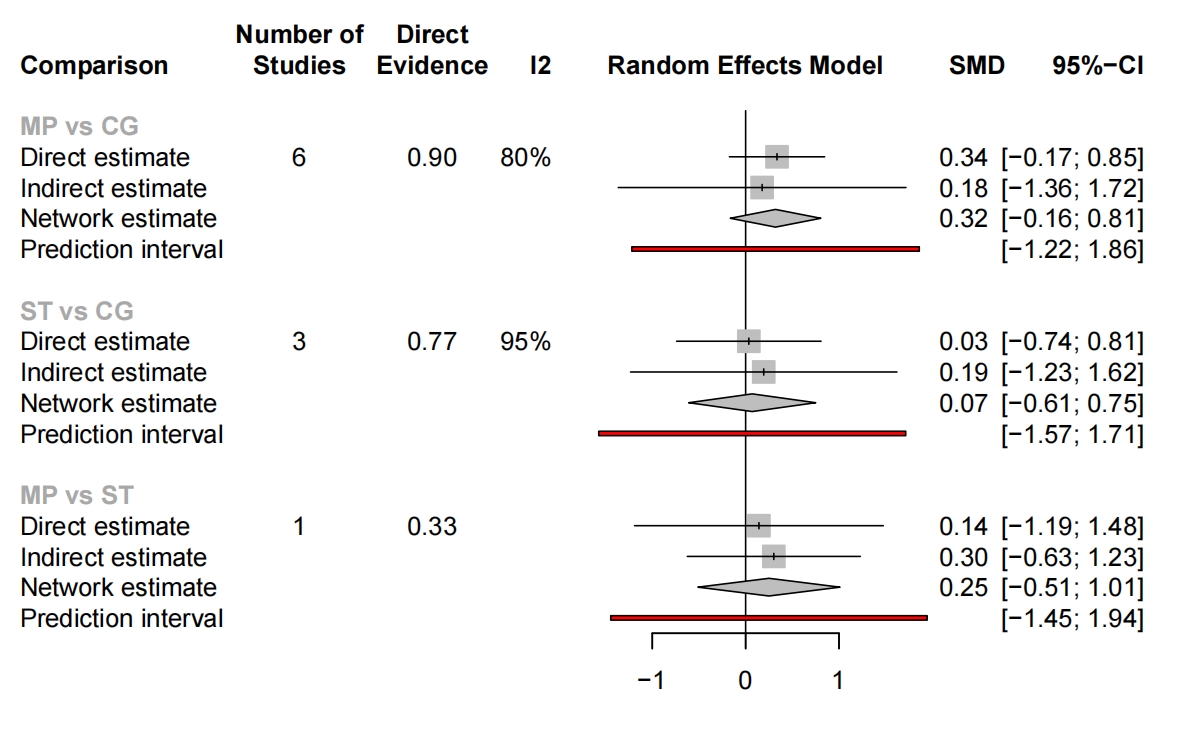
**

**Figures 1–3:** Forest plots showing the effect of different exercise interventions compared with control groups on frailty, activities of daily living, and quality of life in older adults. Using a random-effects model, the figures compare standardized mean differences (SMDs) and their 95% confidence intervals (CIs) between aerobic training (AT), mixed physical training (MP), physical and mental training (PT), strength training (ST), and control groups (CG). The red areas represent the prediction intervals.

**Appendix 8: ‘****Hot spots’ of inconsistency**

**8.1‘Hot Spots’ of Inconsistency: Frailty**

| Legend | |
| --- | --- |
| comparison | - Treatment comparison |
| k | - Number of studies providing direct evidence |
| prop | - Direct evidence proportion |
| nma | - Estimated treatment effect (MD) in network meta- analysis |
| direct | - Estimated treatment effect (MD) derived from direct evidence |
| indir. | - Estimated treatment effect (MD) derived from indirect evidence |
| Diff | - Difference between direct and indirect treatment estimates |
| Z | - z-value of test for disagreement (direct versus indirect) |
| p-value | - p-value of test for disagreement (direct versus indirect) |

Random effects model

| Comparison | k | prop | nma | direct | indir. | Diff | z | p-value |
| --- | --- | --- | --- | --- | --- | --- | --- | --- |
| AT vs CG | 2 | 0.43 | -0.48 | -0.25 | -0.66 | 0.41 | 0.72 | 0.4691 |
| AT vs MP | 2 | 0.46 | 0.15 | 0.11 | 0.17 | -0.06 | -0.11 | 0.9133 |
| AT vs PT | 1 | 0.32 | 0.23 | -0.15 | 0.41 | -0.56 | -0.78 | 0.4329 |
| AT vs ST | 0 | 0 | -0.12 | . | -0.12 |  | . | . |
| MP vs CG | 14 | 0.81 | -0.63 | -0.64 | -0.58 | -0.06 | -0.17 | 0.8653 |
| PT vs CG | 3 | 0.52 | -0.71 | -1.02 | -0.37 | -0.65 | -1.27 | 0.2043 |
| ST VS CG | 9 | 0.76 | -0.37 | -0.29 | -0.62 | 0.33 | 0.73 | 0.4629 |
| MP vs PT | 1 | 0.22 | 0.08 | -0.17 | 0.15 | -0.32 | -0.49 | 0.6260 |
| MP vs ST | 2 | 0.24 | -0.26 | -0.06 | -0.33 | 0.27 | 0.52 | 0.6036 |
| PT vs ST | 1 | 0.24 | -0.34 | -0.11 | -0.42 | 0.31 | 0.44 | 0.6589 |

AT: aerobic training; MP: mixed physical training; PT: physical and mental training; ST: strength training; CG: control group. AT, MP, PT and ST represent different types of exercise interventions, and the specific definitions of each method help clarify their effects in the network meta-analysis. The CG, as a control group, is a benchmark for assessing the effectiveness of other interventions.

**8.2‘Hot Spots’ of Inconsistency: Activities of Daily Living**

| Legend | |
| --- | --- |
| comparison | - Treatment comparison |
| k | - Number of studies providing direct evidence |
| prop | - Direct evidence proportion |
| nma | - Estimated treatment effect (MD) in network meta-analysis |
| direct | - Estimated treatment effect (MD) derived from direct evidence |
| indir. | - Estimated treatment effect (MD) derived from indirect evidence |
| Diff | - Difference between direct and indirect treatment es ti mates |
| Z | - z-value of test for disagreement (direct versus indirect) |
| p-value | - p-value of test for disagreement (direct versus indirect) |

Random effects model

| comparison | k | prop | nma | direct | indir. | Diff | z | p-value |
| --- | --- | --- | --- | --- | --- | --- | --- | --- |
| AT vs CG | 0 | 0 | 0.89 | . | 0.89 | . | . | . |
| AT vs MP | 1 | 0.60 | 0.19 | 0.34 | -0.03 | 0.37 | 0.44 | 0.6598 |
| AT vs PT | 1 | 0.61 | 0.10 | -0.04 | 0.32 | -0.37 | -0.44 | 0.6598 |
| AT vs ST | 0 | 0 | 0.25 | . | 0.25 | . | . | . |
| MP vs CG | 5 | 0.82 | 0.70 | 0.76 | 0.43 | 0.33 | 0.58 | 0.5590 |
| PT VS CG | 2 | 0.63 | 0.79 | 0.83 | 0.73 | 0.10 | 0.15 | 0.8799 |
| ST vs CG | 1 | 0.31 | 0.64 | 0.23 | 0.82 | -0.59 | -0.87 | 0.3842 |
| MP vs PT | 0 | 0 | -0.09 | . | -0.09 | . | . | . |
| MP vs ST | 2 | 0.58 | 0.06 | -0.02 | 0.18 | -0.20 | -0.32 | 0.7498 |
| PT vs ST | 1 | 0.45 | 0.15 | -0.07 | 0.33 | -0.40 | -0.55 | 0.5809 |

AT: aerobic training; MP: mixed physical training; PT: physical and mental training; ST: strength training; CG: control group. AT, MP, PT and ST represent different types of exercise interventions, and the specific definitions of each method help clarify their effects in the network meta-analysis. The CG, as a control group, is a benchmark for assessing the effectiveness of other interventions.

**8.3‘Hot Spots’ of Inconsistency: Quality of Life**

| Legend | |
| --- | --- |
| comparison | - Treatment comparison |
| k | - Number of studies providing direct evidence |
| prop | - Direct evidence proportion |
| nma | - Estimated treatment effect (MD) in network meta-analysis |
| direct | - Estimated treatment effect (MD) derived from direct evidence |
| indir. | - Estimated treatment effect (MD) derived from indirect evidence |
| Diff | - Difference between direct and indirect treatment estimates |
| Z | - z-value of test for disagreement (direct versus indirect) |
| p-value | - p-value of test for disagreement (direct versus indirect) |

Random effects model

| comparison | k | prop | nma | direct | indir. | Diff | z | p-value |
| --- | --- | --- | --- | --- | --- | --- | --- | --- |
| MP vs CG | 6 | 0.90 | 0.32 | 0.34 | 0.18 | 0.16 | 0.19 | 0.8473 |
| PT Vs CG | 0 | 0 | 1.02 | . | 1.02 | . | . | . |
| ST vs CG | 3 | 0.77 | 0.07 | 0.03 | 0.19 | -0.16 | -0.19 | 0.8473 |
| MP VS PT | 0 | 0 | -0.70 | . | -0.70 | . | . | . |
| MP vs ST | 1 | 0.33 | 0.25 | 0.14 | 0.30 | -0.16 | -0.19 | 0.8473 |
| PT vs ST | 1 | 1.00 | 0.95 | 0.95 | . | . | . | . |

AT: aerobic training; MP: mixed physical training; PT: physical and mental training; ST: strength training; CG: control group. AT, MP, PT and ST represent different types of exercise interventions, and the specific definitions of each method help clarify their effects in the network meta-analysis. The CG, as a control group, is a benchmark for assessing the effectiveness of other interventions.

**Appendix 9****:** **Percentage contribution matrices**

**
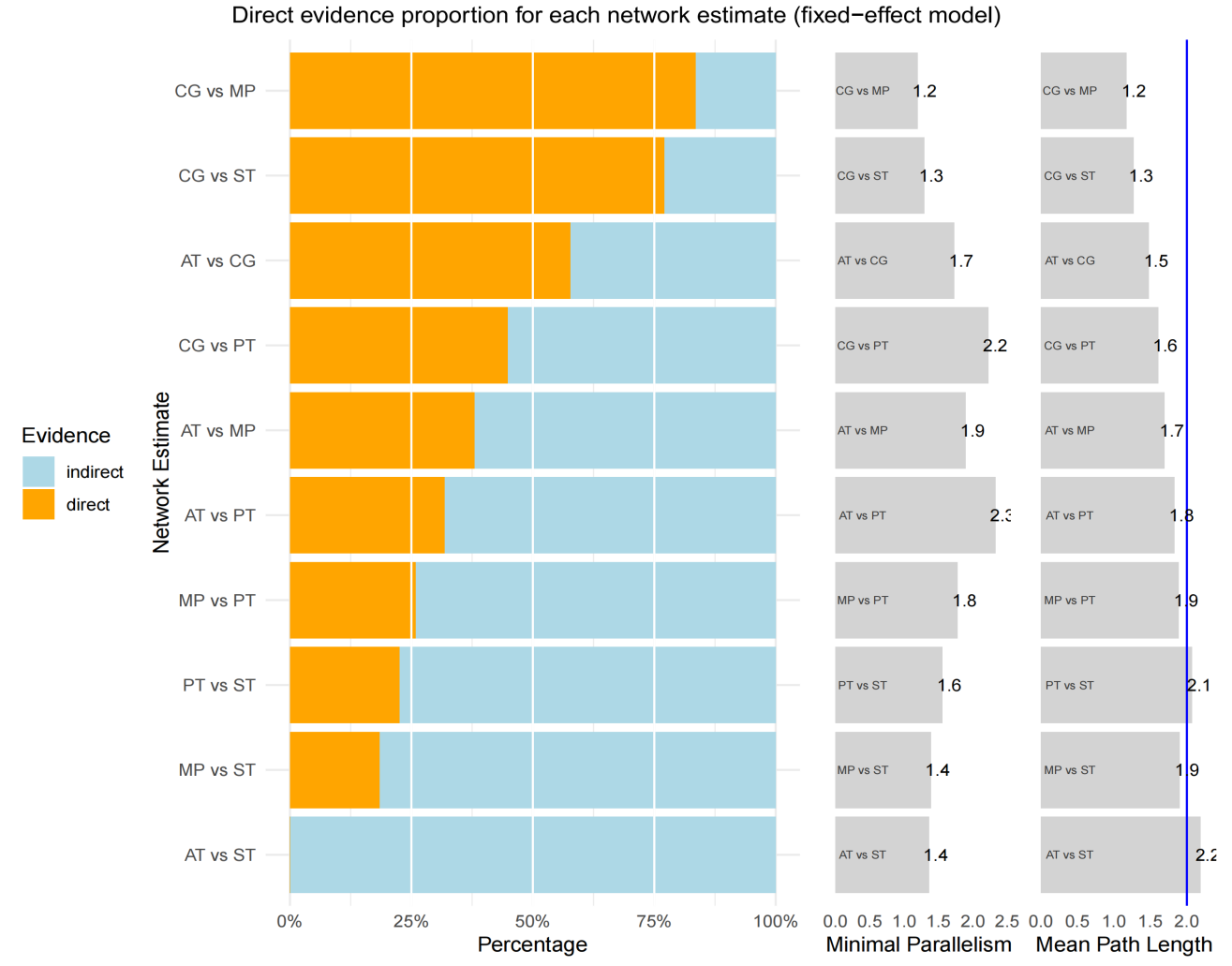
**

**
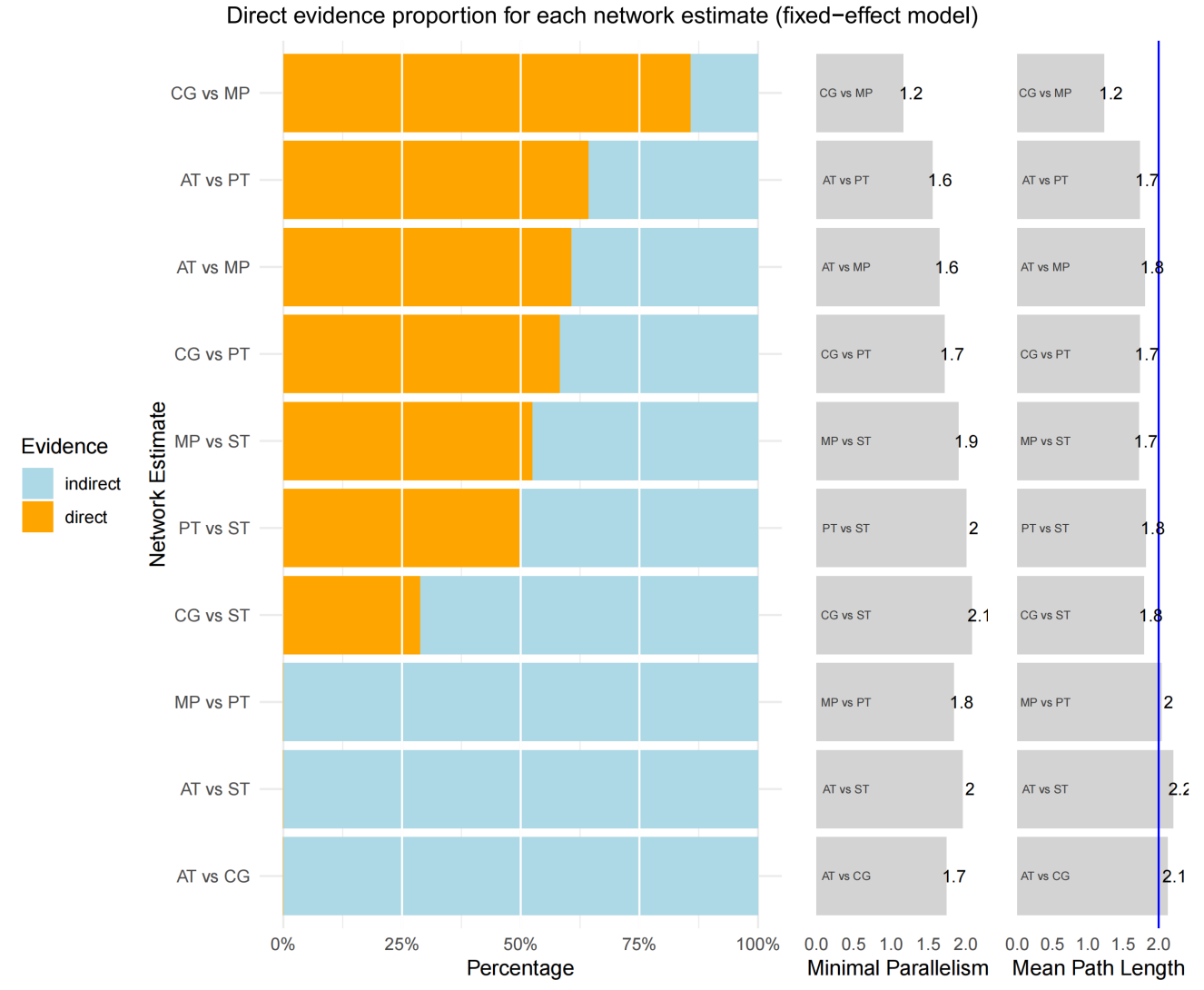

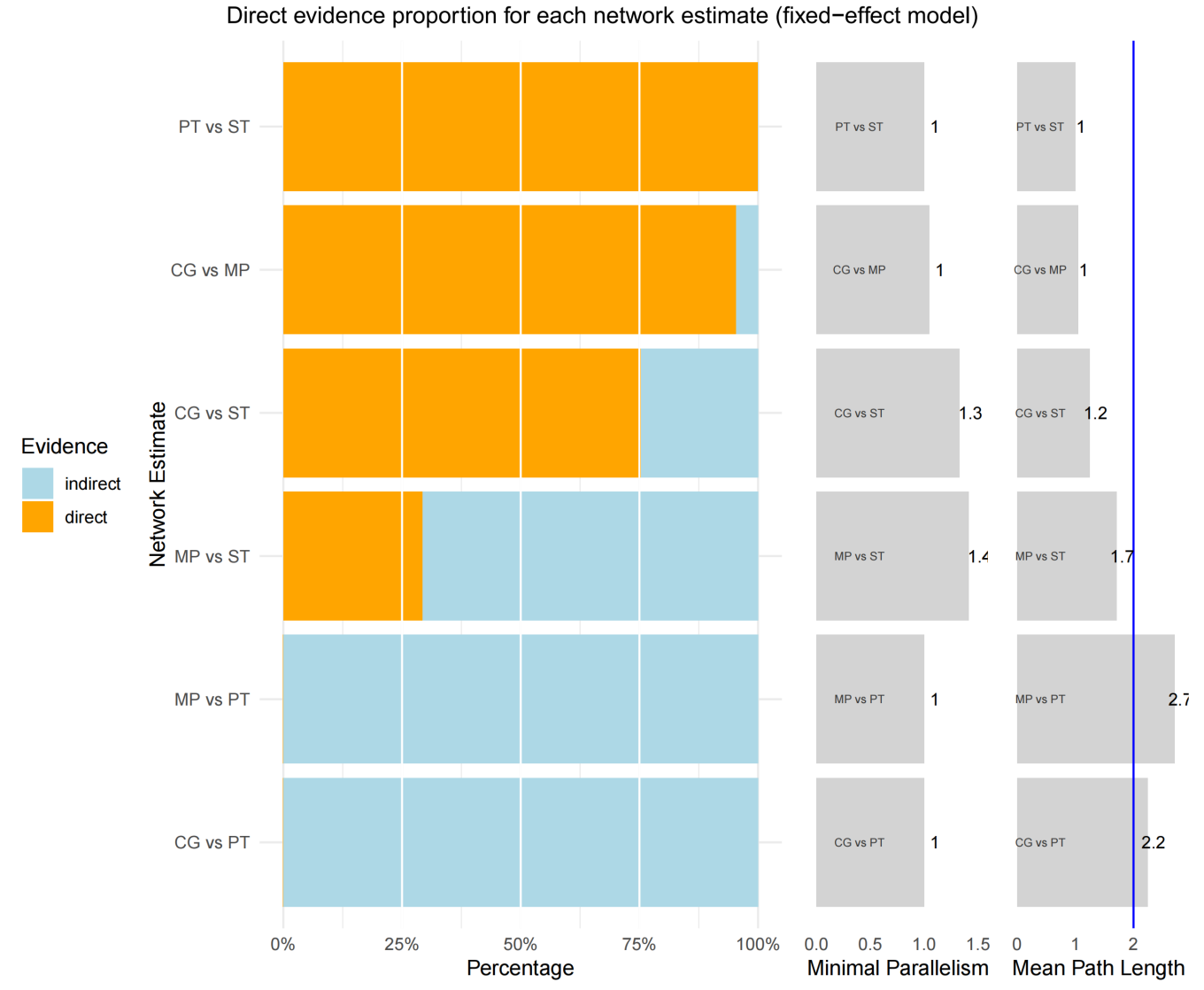
**

**Appendix 10: Funnel plots**

**
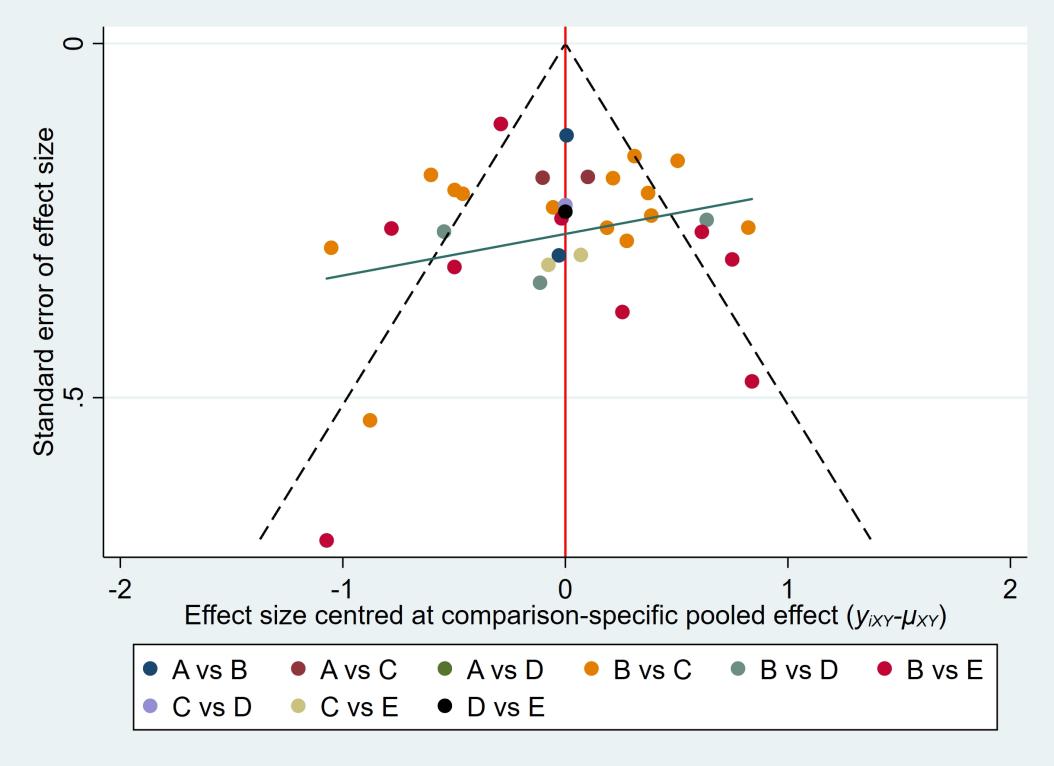
**

**Figure 1:** The funnel plot demonstrates the effect of different exercise interventions on frailty based on the pooled effect center of a specific comparison. A: aerobic training (AT); B: mixed physical training (MP); C: strength training (ST); D: physical and mental training (PT); E: control group (CG). The x-axis of the figure represents the effect size of the pooled effect center for the specific comparison, and the y-axis represents the standard error of the effect size. The dashed area represents the expected distribution of study results in the absence of publication bias. This figure is used to assess whether studies of different exercise interventions for improving frailty are subject to publication bias.

**
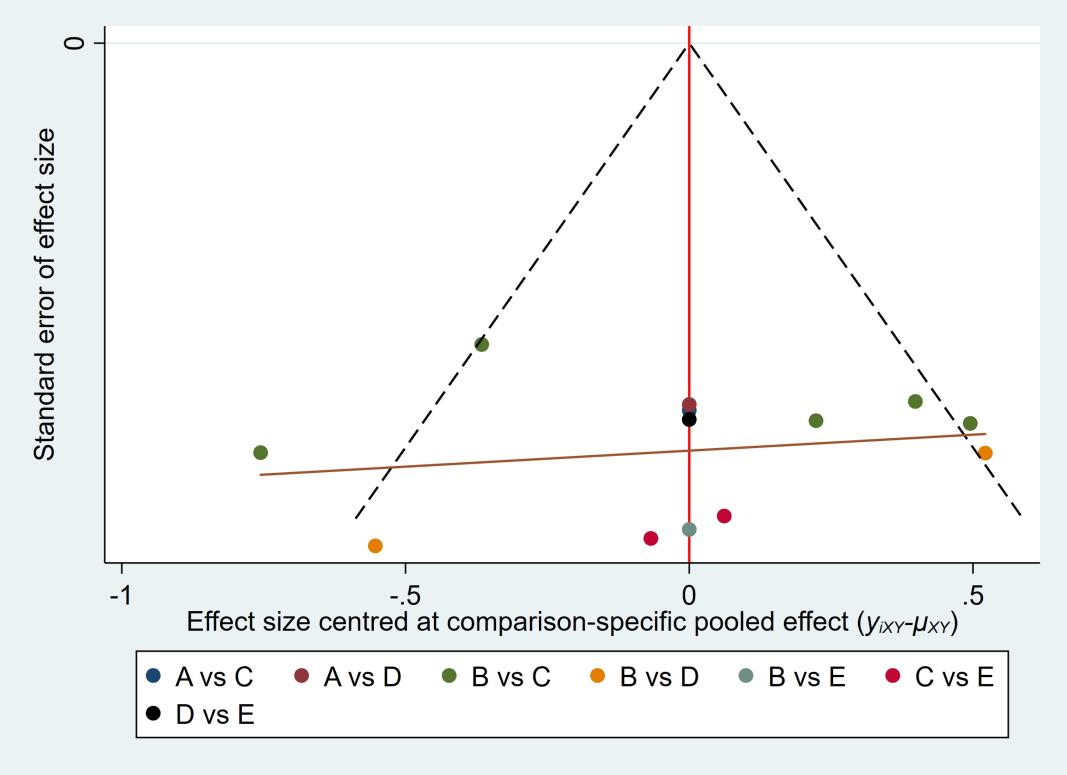
**

**Figure 2:** A funnel plot displays the effect of different exercise interventions on the ability to perform daily activities based on the pooled effect center of a specific comparison. A: aerobic training (AT); B: mixed physical training (MP); C: strength training (ST); D: physical and mental training (PT); E: control group (CG). The x-axis of the figure represents the effect size of the pooled effect center for the specific comparison, and the y-axis represents the standard error of the effect size. The dashed area represents the expected distribution of study results in the absence of publication bias. This figure is used to assess whether studies of different exercise interventions for improving daily activity ability are subject to publication bias.


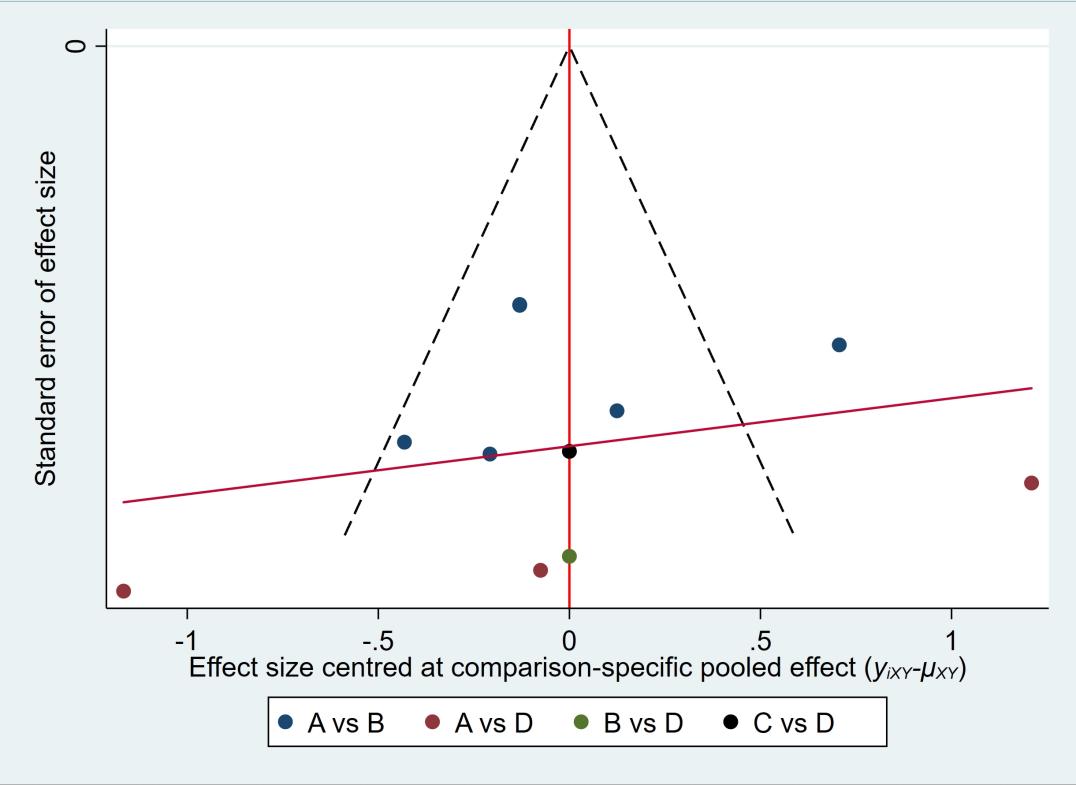


**Figure 3:** Funnel plot showing the effect of different exercise interventions on quality of life, based on the pooled effect center for a given comparison. A: aerobic training (AT); B: mixed physical training (MP); C: strength training (ST); D: physical and mental training (PT). The x-axis of the figure represents the effect size of the pooled effect center for a given comparison, and the y-axis represents the standard error of the effect size. The dashed area represents the expected distribution of study results in the absence of publication bias. This figure is used to assess whether studies on the effect of different exercise interventions on quality of life are subject to publication bias.

**Appendix 11.** **List of included studies**

1. Zech A, Drey M, Freiberger E, Hentschke C, Bauer JM, Sieber CC, et al. Residual effects of muscle strength and muscle power training and detraining on physical function in community-dwelling prefrail older adults: a randomized controlled trial. BMC geriatrics. 2012;12:68.

2. Jose Tarazona-Santabalbina F, Carmen Gomez-Cabrera M, Perez-Ros P, Miguel Martinez-Arnau F, Cabo H, Tsaparas K, et al. A Multicomponent Exercise Intervention that Reverses Frailty and Improves Cognition, Emotion, and Social Networking in the Community-Dwelling Frail Elderly: A Randomized Clinical Trial. Journal of the American Medical Directors Association. 2016 May 1;17(5):426-33.

3. Trombetti A, Hars M, Hsu F-C, Reid KF, Fielding RA, Nelson ME, et al. Effect of physical activity on frailty: Secondary analysis of a randomized controlled trial. Annals of Internal Medicine. 2018;168(5):309-16.

4. Guo JH, Yang D, Zhang J. Effect of individualized multi-exercise intervention on frailty status and somatic function in hospitalized elderly patients. Chinese Journal of Geriatric Multi-Organ Diseases. 2019;18(09):661-4. (In Chinese)

5. Sadjapong U, Yodkeeree S, Sungkarat S, Siviroj P. Multicomponent Exercise Program Reduces Frailty and Inflammatory Biomarkers and Improves Physical Performance in Community-Dwelling Older Adults: A Randomized Controlled Trial. International journal of environmental research and public health. 2020;17(11).

6. Fu R, Zhang R, Liu YL. The effect of a multimodal exercise intervention on the frailty status of older adults in the community. Chinese Geriatric Health Medicine. 2020;18(2). (In Chinese)

7. Jiang CH, Huang T, Song LL, Wan L, Zhang Y, Yang MT. A study to explore the application of exercise exercise in the frailty syndrome of the elderly. Diet Health. 2020(45):285-6. (In Chinese)

8. Tamuleviciute-Prasciene E, Beigiene A, Kubilius R, Thompson MJ, Balne K, Bjarnason-Wehrens B. The impact of additional resistance and balance training in exercise-based cardiac rehabilitation in older patients after valve surgery or intervention: randomized control trial. BMC geriatrics. 2021;21(1):23.

9. Nagai K, Miyamato T, Okamae A, Tamaki A, Fujioka H, Wada Y, et al. Physical activity combined with resistance training reduces symptoms of frailty in older adults: A randomized controlled trial. Archives of gerontology and geriatrics. 2018;76:41-7.

10. Ge YJ, Wu QW, Gao ZP, Xing FM, Liu GT, Wang F et al. Effects of simple-style taijiquan training on the level of debilitation and balance in the pre-frail elderly. Chinese Journal of Gerontology. 2020;40(3). (In Chinese)

11. Yoon DH, Kang D, Kim H-J, Kim J-S, Song HS, Song W. Effect of elastic band-based high-speed power training on cognitive function, physical performance and muscle strength in older women with mild cognitive impairment. Geriatrics and Gerontology International. 2017;17(5):765-72.

12. Yoon DH, Lee JY, Song W. Effects of Resistance Exercise Training on Cognitive Function and Physical Performance in Cognitive Frailty: A Randomized Controlled Trial. The journal of nutrition, health & aging. 2018;22(8):944-51.

13. Chen R, Wu Q, Wang D, Li Z, Liu H, Liu G, et al. Effects of elastic band exercise on the frailty states in pre-frail elderly people. Physiotherapy theory and practice. 2020;36(9):1000-8.

14. Hsieh T-J, Su S-C, Chen C-W, Kang Y-W, Hu M-H, Hsu L-L, et al. Individualized home-based exercise and nutrition interventions improve frailty in older adults: a randomized controlled trial. The international journal of behavioral nutrition and physical activity. 2019;16(1):119.

15. Karssemeijer EGA, Olde Rikkert MGM, Bossers WJR, Sanders LMJ, Aaronson JA, Kessels RPC. Exergaming as a Physical Exercise Strategy Reduces Frailty in People With Dementia: A Randomized Controlled Trial. Journal of the American Medical Directors Association. 2019;20(12):1502.

16. Amasene M，Cadenas-Sanchez C，Echeverria I，et al. Effects of resistance training intervention along with leucine-enriched whey protein supplementation on sarcopenia and frailty in post-hospitalized older adults： preliminary findings of a randomized controlled trial［J］. Clin Med，2021，11（1）：97.

17. Arrieta H，Rezola-Pardo C，Gil SM，et al. Effects of multicomponent exercise on frailty in long-term nursing homes： a randomized controlled trial［J］. J Am Geriatr Soc，2019，67（6）

18. Barrachina-Igual J，Martínez-Arnau FM，Pérez-Ros P，et al. Effectiveness of the PROMUFRA program in pre -frail，community -dwelling older people：a randomized controlled trial［J］. Geriatr Nurs，2021，42（2）：582-591.

19. Caldo-Silva A，Furtado GE，Chupel MU，et al. Effect of training -detraining phases of multicomponent exercises and BCAA supplementation on inflammatory markers and albumin levels in frail older persons［J］. Nutrients，2021，13（4）：1106.

20. Furtado GE，Carvalho HM，Loureiro M，et al. Chair-based exercise programs in institutionalized older women：salivary steroid hormones，disabilities and frailty changes［J］. Exp Gerontol，2020，130：110790.

21. Karssemeijer EGA，Bossers WJR，Aaronson JA，et al. Exergaming as a physical exercise strategy reduces frailty in people with dementia：a randomized controlled trial［J］. J Am Med Dir Assoc，2019，20（12）： 1502-1508.e1.

22. Liao YY，Chen IH，Wang RY. Effects of Kinect-based exergaming on frailty status and physical performance in prefrail and frail elderly：a randomized controlled trial［J］. Sci Rep，2019，9：9353.

23. Liu T，Wang C，Sun J，et al. The effects of an integrated exercise intervention on the attenuation of frailty in elderly nursing homes：a cluster randomized controlled trial［J］. J Nutr Health Aging，2022，26 （3）：222-229.

24. Meng NH，Li CI，Liu CS，et al. Effects of concurrent aerobic and resistance exercise in frail and pre-frail older adults：a randomized trial of supervised versus home-based programs［J］. Medicine，2020， 99（29）：e21187.

25. Rezola-Pardo C，Arrieta H，Gil SM，et al. Comparison between multicomponent and simultaneous dual-task exercise interventions in longterm nursing home residents：the Ageing-ONDUAL-TASK randomized controlled study［J］. Age Ageing，2019，48（6）：817-823.

26. Swales B，Ryde GC，Whittaker AC. A randomized controlled feasibility trial evaluating a resistance training intervention with frail older adults in residential care：the keeping active in residential elderly trial［J］. J Aging Phys Act，2022，30（3）：364-388.

27. Chen RJ. The Effect of Elastic Band Exercise on Frailty Status and Quality of Life in Pre-frail Elderly People［D］. TangShan：North China University of Science and Technology，2019.

28. Hou XL. Research of the Application of Baduanjin in Institutionalized Elderly Frailty［D］. Chengdu： Chengdu University of traditional Chinese Medicine，2018.

29. Huang HY， Dai X，Hu R，et al. Effects of multicomponent training on nutritional status and muscle function in frail elderly adults［J］. Chin J Prac Nurs，2022，38（7）：530-536.

30. Liu CR. Analysis of the effect of aerobic exercise on the improvement of weakness in the elderly［D］. Qingdao：Qingdao University，2015.

31. Zhang HJ，Tang LM，Sun WG，et al. Application of Baduanjin combined with resistance exercise in rural elderly［J］. Chin J Mod Nurs，2021，27（26）：3604-3608.

32. Zheng YJ. Effects of Multi-component Exercise Program on Frailty and Physical Functions in Pre-frail Older Adults:a Randomized Controlled Trial［D］. Guangzhou：Southern Medical University，2020.

33. Zhang X, Van Der Schans C P, Liu Y, et al. Efficacy of dance intervention for improving frailty among chinese older adults living in the community: A randomized controlled trial[J]. Journal of Aging and Physical Activity, 2023, 31(5): 806-814.

34. Lin Y-J, Hsu W-C, Wang K C, et al. Interactive boxing–cycling on frailty and activity limitations in frail and prefrail older adults: A randomized controlled trial[J]. Annals of physical and rehabilitation medicine, 2024, 67(4): 101819.

35. Tuan S-H, Chang L-H, Sun S-F, et al. Assessing the Clinical Effectiveness of an Exergame-Based Exercise Training Program Using Ring Fit Adventure to Prevent and Postpone Frailty and Sarcopenia Among Older Adults in Rural Long-Term Care Facilities: Randomized Controlled Trial[J]. Journal of Medical Internet Research, 2024, 26: e59468.
